# Computational Insights into the Inhibitory Mechanism of Human AKT1 by an Orally Active Inhibitor, MK-2206

**DOI:** 10.1371/journal.pone.0109705

**Published:** 2014-10-17

**Authors:** Mohd Rehan, Mohd A. Beg, Shadma Parveen, Ghazi A. Damanhouri, Galila F. Zaher

**Affiliations:** 1 King Fahd Medical Research Center, King Abdulaziz University, Jeddah, Kingdom of Saudi Arabia; 2 Bareilly College, M. J. P. Rohilkhand University, Bareilly, Uttar Pradesh, India; 3 Department of Haematology, Faculty of Medicine, King Abdulaziz University, Jeddah, Kingdom of Saudi Arabia; Hormel Institute, University of Minnesota, United States of America

## Abstract

The AKT signaling pathway has been identified as an important target for cancer therapy. Among small-molecule inhibitors of AKT that have shown tremendous potential in inhibiting cancer, MK-2206 is a highly potent, selective and orally active allosteric inhibitor. Promising preclinical anticancer results have led to entry of MK-2206 into Phase I/II clinical trials. Despite such importance, the exact binding mechanism and the molecular interactions of MK-2206 with human AKT are not available. The current study investigated the exact binding mode and the molecular interactions of MK-2206 with human AKT isoforms using molecular docking and (un)binding simulation analyses. The study also involved the docking analyses of the structural analogs of MK-2206 to AKT1 and proposed one as better inhibitor. The Dock was used for docking simulations of MK-2206 into the allosteric site of AKT isoforms. The Ligplot+ was used for analyses of polar and hydrophobic interactions between AKT isoforms and the ligands. The MoMa-LigPath web server was used to simulate the ligand (un)binding from the binding site to the surface of the protein. In the docking and (un)binding simulation analyses of MK-2206 with human AKT1, the Trp-80 was the key residue and showed highest decrease in the solvent accessibility, highest number of hydrophobic interactions, and the most consistent involvement in all (un)binding simulation phases. The number of molecular interactions identified and calculated binding energies and dissociation constants from the co-complex structures of these isoforms, clearly explained the varying affinity of MK-2206 towards these isoforms. The (un)binding simulation analyses identified various additional residues which despite being away from the binding site, play important role in initial binding of the ligand. Thus, the docking and (un)binding simulation analyses of MK-2206 with AKT isoforms and its structure analogs will provide a suitable model for studying drug-protein interaction and will help in designing better drugs.

## Introduction

The PI3K/AKT/mTOR signaling pathway is an important pathway for normal cellular functions in the human body and is the most commonly dysregulated pathway in cancer [1], [2]. The AKT is one of the key proteins of this pathway belonging to the serine/threonine AGC protein kinase family and is also known as Protein Kinase B (PKB). The human AKT is found in three isoforms AKT1, 2, and 3, also known as PKB-α, -β and -γ and these isoforms are highly homologous multi-domain proteins possessing both common and distinct cellular functions [3], [4]. The AKT is involved in several functions in the body such as metabolism, growth, proliferation, differentiation, and survival of the cells [5], [6]. Conversely in regards to cancer, the constant activation and/or over-expression of AKT frequently contributes to the resistance to cancer chemotherapy or radiotherapy [7], [8]. Recently, in vitro and in vivo studies with small molecule inhibitors of the AKT have been successful in attenuating chemotherapeutic resistance when combined with the standard chemotherapy [9], [10]. Therefore, specific inhibition of AKT activity may be a good alternative approach to treat cancer and increase the efficacy of chemotherapy. In this regard, significant efforts have been made to generate chemical compounds designed specifically to target AKT or other targets in the AKT signaling pathway and some of these compounds are in clinical trials for cancer treatment [2]. The majority of known AKT inhibitors are ATP competitive and have poor specificity against other closely related kinases. The increasing attention for AKT specific inhibitors or even AKT-isoform specific inhibitors led to the discovery of allosteric AKT inhibitors [11]–[13]. One such compound, MK-2206 (IUPAC name: 8-[4-(1-aminocyclobutyl)phenyl]-9-phenyl-2H-[1], [2], [4]triazolo[3,4-f][1], [6]naphthyridin-3-one), is a highly potent, selective, and orally active allosteric inhibitor of AKT which has been recently identified [14]–[16] and is effective at nanomolar concentration against purified recombinant human AKT1, 2, and 3 [17]. The compound is almost equally potent for human AKT1 and human AKT2 (IC_50,_ 5 nmol/L and 12 nmol/L, respectively) and is about five-fold less potent against human AKT3 (IC_50_, 65 nmol/L) [17]. Various preclinical studies have demonstrated that MK-2206 effectively inhibited AKT and promoted cancer cell death when used alone or augmented the efficacy of several anti-cancer agents when used in combination [14]–[15], [18]–[22]. The MK-2206 is orally active and has been shown to be safe in humans [23]–[24]. The preclinical results with this compound were highly successful and it is now in phase I/II clinical trials for treatments of solid tumors and acute myelogenous leukemia (http://clinicaltrials.gov/ct2/results?term=MK2206).

There is conclusive evidence that MK-2206 is a promising compound for inclusion in the standard cancer therapy protocols. Several studies have shown the MK-2206 mediated inhibition of the key cancer-regulatory-protein AKT, however, the exact binding mechanisms and the molecular interactions of MK-2206 with AKT have not been studied. Recently [25], a molecular dynamic simulation study of MK-2206 was performed to calculate the binding free energy at the binding site of AKT1. The modeling involved sketching of MK-2206 at the AKT1 binding site by modifying the structure of bound Inhibitor VIII in the co-complex structure. However, this model may not correctly predict the binding mode of MK-2206 as it is not a derivative of Inhibitor VIII. In this regard, the present study was proposed to investigate the structural and molecular details of MK-2206 binding against AKT1 using molecular docking and ligand (un)binding simulation approach. In the current study we used a molecular docking program which takes into account the shape complementarity, and van der Walls and Coulombic electrostatic energies at the binding site to identify the correct pose of MK-2206. We also validated the MK-2206 binding mode with the previously existing knowledge of key interacting residue in allosteric inhibition of AKT1. Therefore, we believe that our modeling provides better representation of the binding mode and gives more accurate prediction of molecular interaction of MK-2206 with AKT1. Further, the homology modeling and docking analyses with the AKT2 and AKT3 were also carried out to explain the varying affinity of MK-2206 towards these isoforms. Finally, we also performed docking study of structure analogs of MK-2206 to AKT1 and proposed an structural analog as a better inhibitor. This study will shed light on inhibitory mechanism of human AKT isoforms by MK-2206 and its structure analogs, and will help experimental biologist in testing and designing better inhibitors.

## Materials and Methods

### Data retrieval

The molecular structure of MK-2206 was retrieved from PubChem compound database (CID, 24964624). The 3-D structure of human AKT1 was obtained from Protein Data Bank (PDB, http://www.rcsb.org/; PDB ID: 3O96). This structure is a co-complex structure containing an allosteric inhibitor, Inhibitor VIII. This AKT1 structure (PDB ID: 3O96) was chosen as our study involved the docking of the allosteric inhibitor, MK-2206 to AKT1 and required a clue for allosteric site from the bound allosteric inhibitor. Further, the information of the bound inhibitor can be used to compare the binding of the docked inhibitor. The amino acid sequences for all three isoforms AKT1, AKT2, and AKT3 were obtained from UniProtKB/Swiss-Prot (http://www.uniprot.org/; IDs: P31749, P31751, Q9Y243, respectively).

### Structural analogs of MK-2206

To retrieve structural analogs of MK-2206, a search was performed using option “Similar Compounds” in the PubChem. The “Similar Compound” search involves calculation of the Tanimoto coefficient which requires PubChem dictionary-based binary fingerprint (https://pubchem.ncbi.nlm.nih.gov/search/help_search.html). The fingerprint consists of series of chemical substructure “keys” and each key (in binary form) indicates the presence or absence of a particular substructure in a compound. Thus, the binary keys together form a “fingerprint” of a particular chemical compound. The fingerprints do not take into consideration the variation in stereochemical or isotopic information. The Tanimoto coefficient measures the degree of similarity and a threshold value is set to retrieve the compounds similar to a query structure. A threshold of “100%” refers to “exact match” (ignoring stereo or isotopic information), whereas a threshold of “0%” would return all compounds present in PubChem database. The threshold utilized for “Similar Compounds” search of MK-2206 was the pre-programmed default (80%), which retrieved 45 similar compounds. These compounds further on filtering using Lipinski's Rule-of-five (for evaluating druglikeness of compounds) shortlisted to 33 compounds. On visual analyses, irrelevant and redundant structures were removed and, finally 30 of 33 structures were selected for further study.

### Homology modeling of human AKT2 and AKT3

The 3-D structures of human AKT2 and AKT3 which were available in PDB were truncated versions. In order to model the full proteins, Modeller9v11 package [26] was used. The templates were identified using ‘blastp’ against PDB database. A close homologous structure of human AKT1 with PDB Id: 3O96 was identified for AKT2 (83% identity, 93% similarity) and AKT3 (83% identity, 90% similarity) both. This structure of human AKT1 (PDB Id: 3O96) is same which we selected for docking and (un)binding simulation analyses. In addition to the common template, the truncated AKT2 structure (partial kinase domain) covering 143–481 residues (PDB Id: 1MRY) and the truncated AKT3 structure (Ph domain) covering 1–118 residues (PDB Id: 2×18) were also considered while modeling AKT2 and AKT3 proteins respectively. A total of 100 three-dimensional models were generated and best 5 models were picked in each case. The selection of best 5 structure models out of 100 generated models was performed on the basis of lower value of the Modeller objective function or the DOPE assessment score and with the higher value of GA341 assessment score. To evaluate and select the single best model, steriochemical properties of the five best models were assessed using PROCHECK [27].

### Molecular docking

Dock v.6.5 (University of California, San Francisco) was used for docking simulations of MK-2206 into the allosteric site of AKT1 [28]. The best docked conformation search strategy used was Random Conformation Search which utilizes the grid-based scoring functions of Coulombic and Lennard-Jones forces. Chimera v.1.6.2 [29] was used in the structure preparation of the protein and the ligand initially required by Dock and also in visualizing the structures at various stages of docking process.

### Analyses of docked protein-ligand complex

To generate an illustration and analyze the whole protein-ligand complex, PyMOL v.1.3 was used [30]. For the polar and hydrophobic interactions between AKT1 and the ligand MK-2206, illustrations were generated and the analyses were performed by Ligplot+ v.1.4.3 program [31]–[32]. For further confirmation and calculating the extent of involvement of interacting residues obtained from Ligplot+, loss in ASA (Accessible Surface Area) was evaluated after the MK-2206 binding to AKT1. It is known that for a residue to be involved in interaction, it should lose more than 10 Å^2^ ASA in the direction from unbound to the bound state [33]. The ASA calculations of unbound protein and the protein-ligand complex were performed by Naccess v.2.1.1 [34]. The loss in ASA, ΔASA of the *i* ^th^ residue in the direction from unbound to bound state was calculated using the expression:
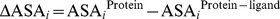



In addition to the Dock score (Grid score) obtained from Dock v.6.5 [28], the binding energy and dissociation constants were also calculated using X-Score v.1.2.11 [35]–[36].

### Protein-ligand (un)binding simulation

To simulate the ligand (un)binding from the binding site to the surface of the protein, a Molecular Motion Algorithms (MoMA) based web server, MoMa-LigPath (http://moma.laas.fr), was used [37]–[38]. The MoMa-LigPath takes into consideration the flexibility for the protein side-chains and the ligand and involves geometric constraints only. The program simulates how the ligand is driven to the binding site from the surface of the protein or from the binding site to the surface. The program also provides snapshots of molecular interactions bringing the ligand from the surface of the protein to the binding site. During the process of (un)binding simulation, the program also identifies the important residues of the target protein which despite being away from the binding site, still help in driving the ligand to the binding site of the protein.

### Protein sequence alignment and analyses

The amino acid sequences of three AKT isoforms were aligned using Muscle v.3.8.31 [39], and further analyses and illustration were prepared by Jalview v.2.8 [40]–[41].

## Results and Discussion

### Molecular docking analyses of MK-2206

**Figure 1:**
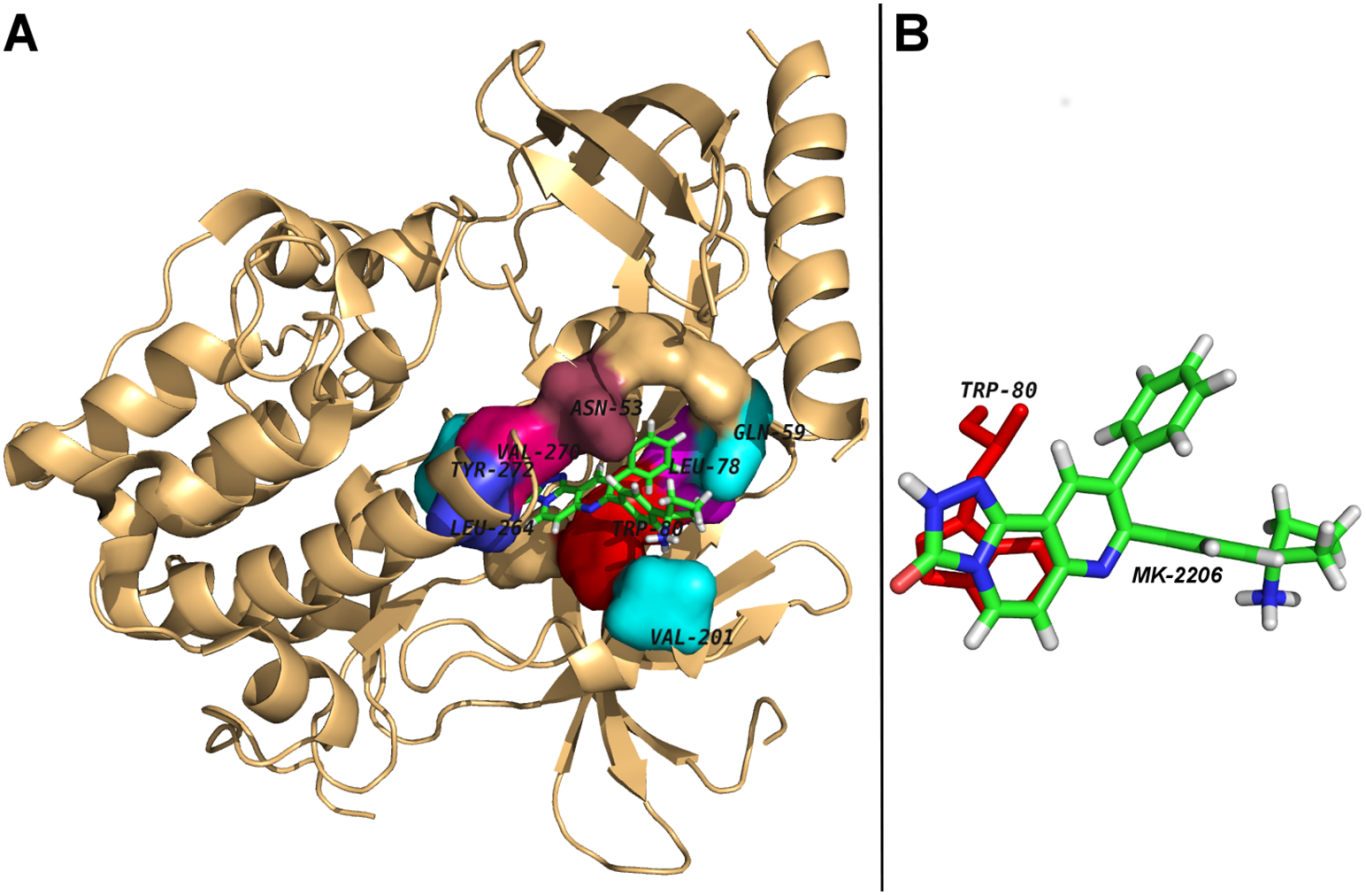
Molecular docking analyses of MK-2206 to the allosteric site of human AKT1. Panel A: Human AKT1 is illustrated in cartoon representation and MK-2206 is in stick representation. The interacting residues are labeled and are shown as surface in different colors. Panel B: The possible aromatic stacking interaction of the amino acid residue, Trp-80 through its indole group with naphthyridin moiety of MK-2206 is shown.

**Figure 2:**
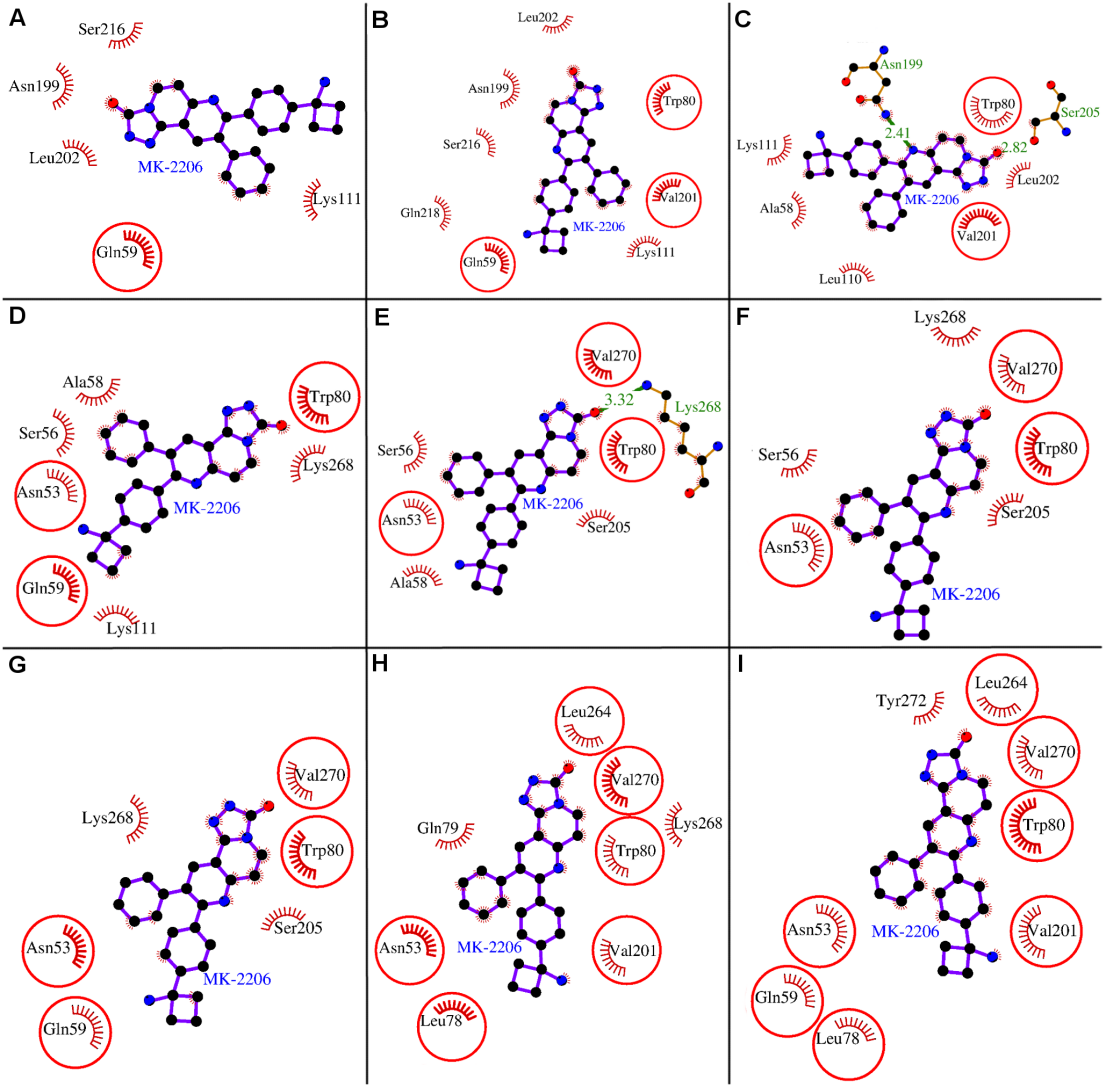
(Un)binding simulation analyses of MK-2206 binding to the allosteric site of human AKT1. Panels A–I: The (un)binding simulation phases of MK-2206; ‘A’ denotes farthest phase from the binding site, ‘H’ - the closest to the binding site, and ‘I’ - the binding site phase. The hydrogen bonds are shown as green-dashed lines with indicated bond length and the residues involved in hydrophobic interactions are shown as red arcs. The residues which are common to the last phase (F) are encircled.

The docking analyses of MK-2206 revealed that the compound packed against the residues Asn-53, Gln-59, Leu-78, Trp-80, Val-201, Leu-264, Val-270, and Tyr-272 of AKT1 and was stabilized by the hydrophobic interactions (Fig. 1A). The Dock score was negative with high absolute value and number of hydrophobic interactions that kept MK-2206 bound in the cavity was also reasonably high (25 interactions from 8 different residues, Table 1). All identified MK-2206 interacting residues of AKT1 with the loss in solvent accessibility and the total number of hydrophobic interactions are listed in Table 1. The higher the loss in solvent accessibility for a residue in the direction from unbound to the bound state, the more involved is the residue in the ligand binding [33]. The importance of AKT1 residues for MK-2206 binding were also ranked on the basis of loss in solvent accessibility (Table 1). The Trp-80 was identified as the key residue of AKT1 and was involved in the majority of hydrophobic interactions and showed highest decrease in its solvent accessibility after MK-2206 binding (approx. 77.85 Å^2^) as shown (Table 1). The Trp-80 was also the most common residue through all phases of MK-2206 (un)binding simulation (Fig. 2), demonstrating its importance in initial binding and finally bringing the drug into the active site of AKT1. Furthermore, Trp-80 also seemed to make aromatic stacking interactions between the indole group and naphthyridin moiety of MK-2206 (Fig. 1B). All the findings of Trp-80 as key interacting residue validates MK-2206 binding mode as these findings were consistent with a previous study [42] in which it is shown that the inhibition of AKT1 by Akti (an allosteric inhibitor of AKT) is critically dependent upon a solvent-exposed tryptophan residue (Trp-80) present in all three AKT isoforms and whose mutation to alanine yields an Akti-resistant kinase.

**Table 1:** The human AKT1 residues interacting with MK-2206 are listed with the number of non-bonding contacts and the loss in Accessible Surface Area (ASA).

Interacting residues	No. of hydrophobic contacts	ΔASA (Å^2^)
Asn-53	3	48.65^2^
Gln-59	1	16.44^7^
Leu-78	1	5.61^8^
Trp-80*	13	77.85^1^
Val-201	3	38.37^3^
Leu-264	1	19.16^5^
Val-270	2	35.8^4^
Tyr-272	1	16.61^6^

The ranking of residues on the basis of loss in solvent accessibility is indicated by superscripts with the value of ΔASA.

*The most common residue in all phases of (un)binding simulation.

### Human AKT isoforms and comparison of interacting residues for MK-2206

**Figure 3:**
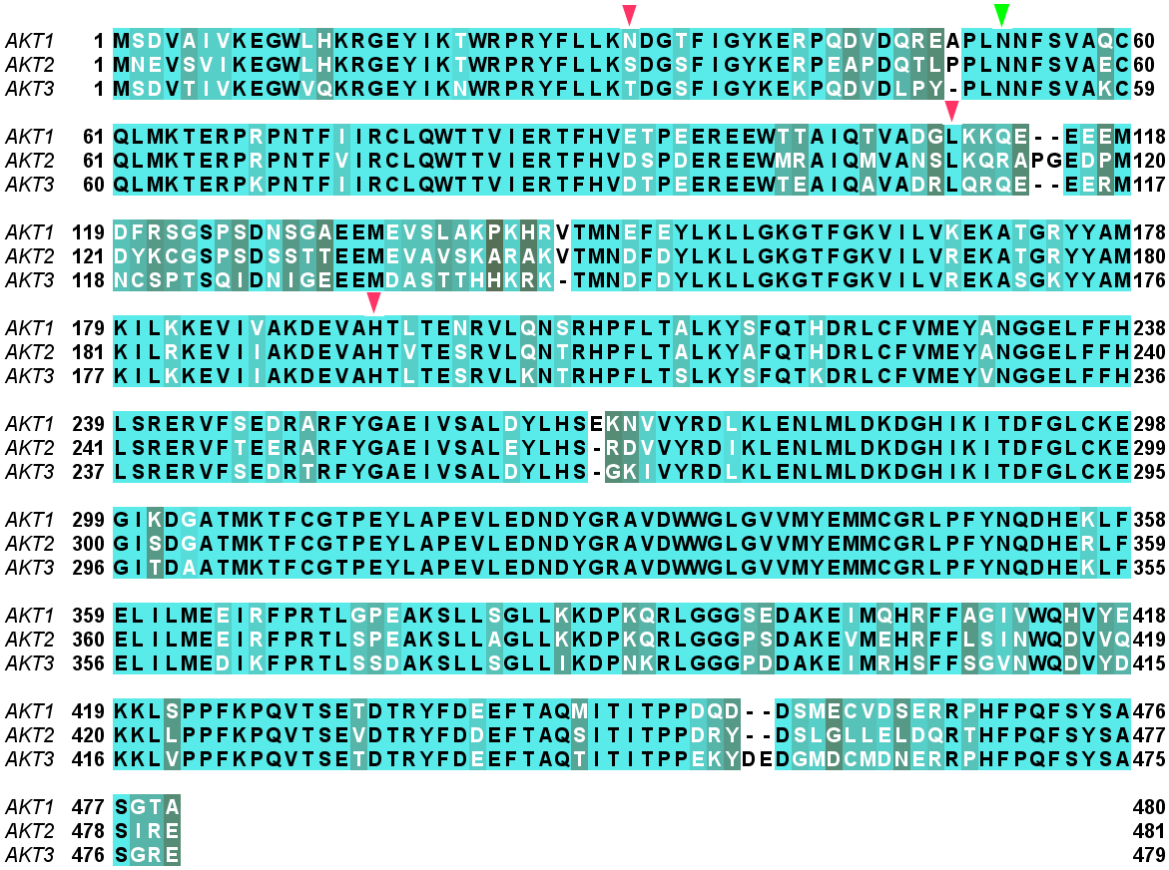
Multiple Sequence Alignment of the three AKT isoforms, AKT1, AKT2, and AKT3. The conserved positions are shown in light green and the corresponding amino acids in black font, whereas the less-conserved positions are shown in gray color with the corresponding amino acids in white font. The initial and final position of each isoform in all the rows of the alignment is also provided. The position-equivalent-residues (residues of different isoforms falling at same column position in the isoform alignment) overlapping among the interacting residues of MK-2206 are marked by triangles; the green triangle (Asn-53) indicates the residue overlapping between AKT1 and AKT2 binding, while the red triangles indicate the position-equivalent-residues overlapping among the interacting residues of AKT2 and AKT3 which are Ser-31, Leu-110, and His-196 of AKT2 (corresponding to Thr-31, Leu-109, and His-192 of AKT3 respectively).

**Figure 4:**
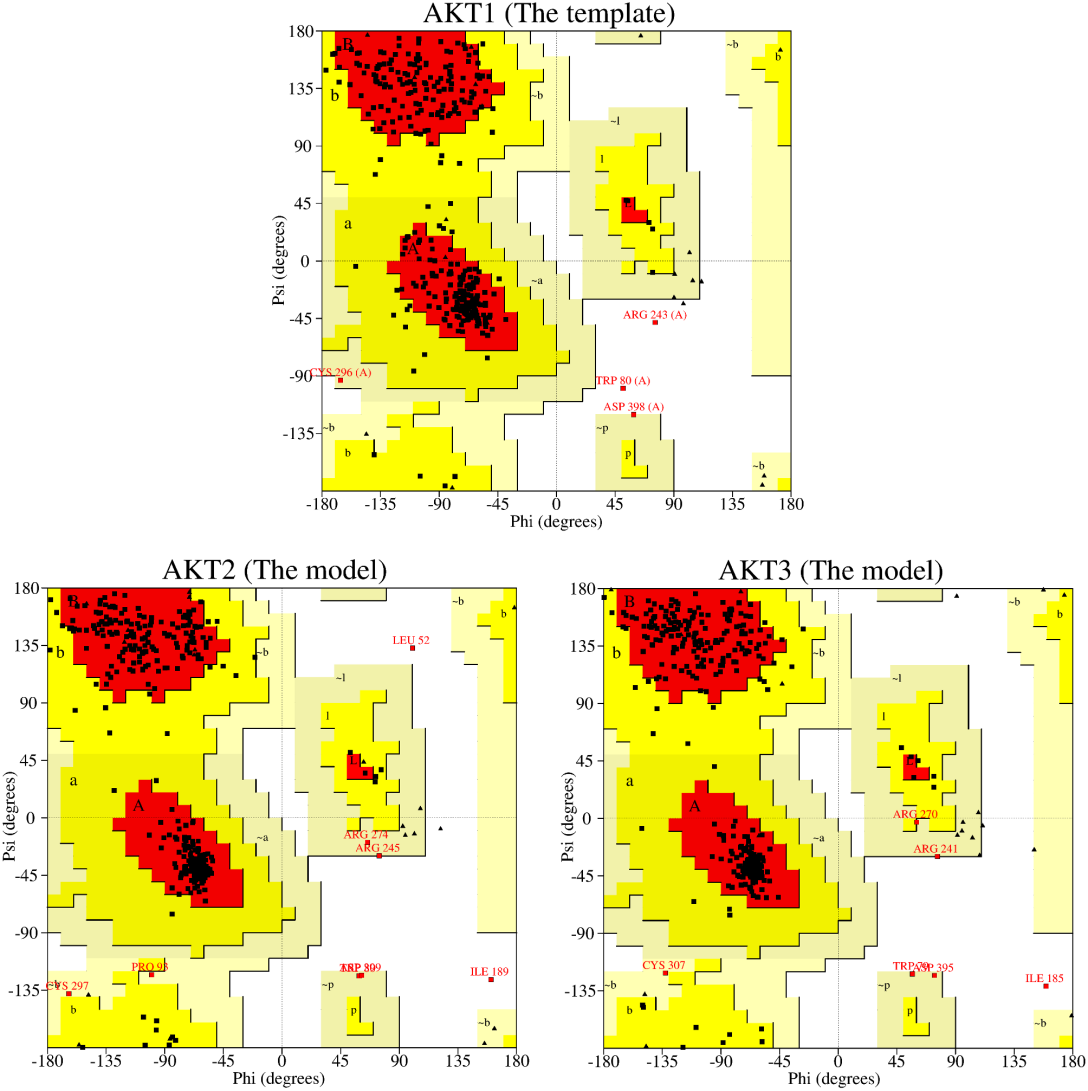
Ramachandran plot showing the residues as square dots lying in the four different regions, most favorable, additional allowed, generously allowed, and disallowed regions.

All the three human AKT isoforms showed a high degree of amino acid sequence homology (Fig. 3). The calculated percentage identity of AKT1 with AKT2 and AKT3 was 81.12% and 82.37% respectively. The 3-D structure models of AKT2 and AKT3 were generated as described in Materials and Methods section. On evaluation by Ramachandran plot, the resultant models (Fig. 4) revealed that there are few residues in generously allowed and disallowed regions (Table 2). All the criteria including the high percentage of residues in allowed regions of the Ramachandran plot, DOPE energy profile comparison with the template, and less numbers of labeled residues (unfavorable conformation) deduced from Ramachandran (Fig. 4, Table 2) and Chi1-Chi2 plots provide confidence to the models. In essence, we found that the models generated were of good quality and can effectively be used for further studies.

**Table 2:** Procheck analyses for quality of structure models of AKT2 and AKT3 with the common template.

	Ramachandran Plot Analyses				Labeled residues	
Protein	Most favorable	Additional Allowed	Generously Allowed	Disallowed	All Ramachandrans	Chi1-Chi2
Template	87.0%	11.7%	0.6%	0.6%	12 (out of 355)	9 (out of 252)
AKT2	89.0%	9.0%	1.4%	0.6%	15 (out of 389)	8 (out of 263)
AKT3	88.8%	9.5%	1.4%	0.3%	18 (out of 391)	3 (out of 266)

Ramachandran plot analyses showing the percentage of residues lying in each of the four different regions. In disallowed region, the number of residues is also given in parantheses with percentage. The number of labeled residues in all Ramachandrans and Chi1–Chi2 are also given in the table.

**Figure 5:**
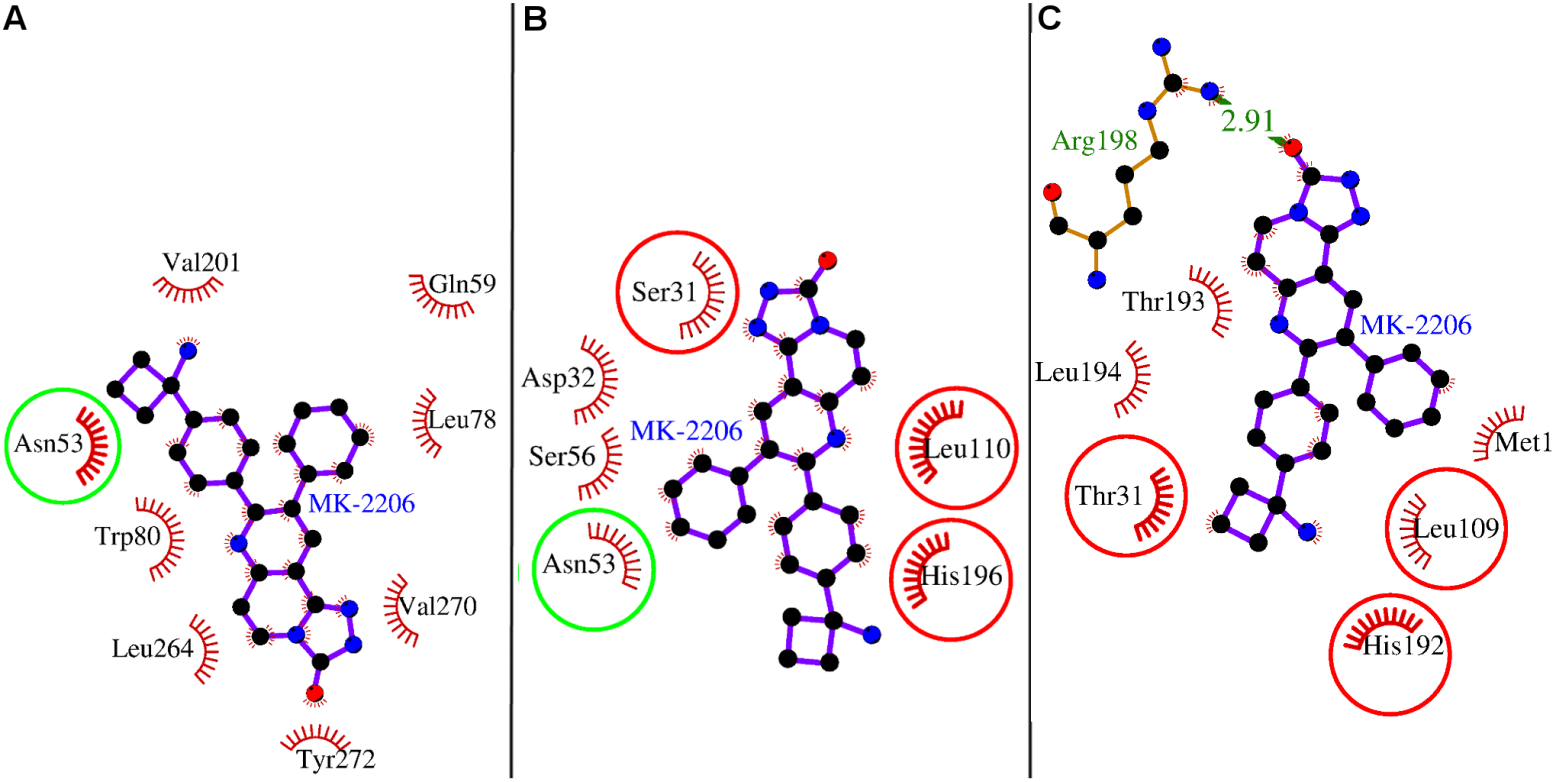
Comparison of MK-2206 binding and interacting residues of all three isoforms of AKT. Only one residue Asn-53 was shared among interacting residues of AKT1 and AKT2, and shown as encircled in green. Three position-equivalent-residues (residues of different isoforms falling at same column position in the isoform alignment) were shared among interacting residues of AKT2 and AKT3, and shown as encircled in red.

When MK-2206 was docked to the structure models of AKT2 and AKT3, we found its binding mode was different in different isoforms. This may be observed because of change in conformation induced by variation in amino acids for these isoforms as shown in the isoform alignment (Fig. 3). However, there were some position-equivalent-residues (residues of different isoforms falling at same column position in the isoform alignment) among the interacting residues of these isoforms which were overlapping (Fig. 3). As shown in Fig. 5, the interacting residues of the isoforms AKT1 and AKT2 shared only one residue Asn-53 as common. Whereas, in case of AKT2 and AKT3, three position-equivalent-residues (residues of the isoforms falling at same column position in the isoform alignment, Fig. 3) were common viz. Ser-31, Leu-110, and His-196 of AKT2 (corresponding to Thr-31, Leu-109, and His-192 of AKT3 respectively) as displayed in Fig. 5. This showed AKT1 shared binding site with AKT2 but not with AKT3 within the allosteric site and AKT3 shared binding site with AKT2 but not with AKT1. To sum up, the isoform AKT2 is sharing binding site with AKT1 and AKT2 both but it is doing so through different overlapping position-equivalent-residue pairs. When we looked at the molecular-interactions and interacting residues of these isoforms, we found that the number of hydrophobic interactions of AKT1 and AKT2 was similar (25 interactions, AKT1; 24 interactions, AKT2) but it decreased to a higher degree for AKT3 (11 interactions) as shown in Table 3–4. We also calculated binding energies and dissociation constants for the co-complex structures of these isoforms (Table 5). We found that the binding energy order was AKT1 at highest, then AKT2, followed by AKT3 with greater difference. Furthermore, the dissociation constant of AKT1 was also slightly more than that of AKT2 but it was ten times of AKT3. These findings of the number of molecular interactions, the binding energy, and the dissociation constant were corroborating with one another and in agreement with what is reported in literature [17] that the binding affinity of MK-2206 is less for AKT2 with respect to that of AKT1 but decreased to a higher degree for AKT3.

**Table 3:** The human AKT2 residues interacting with MK-2206 are listed with the number of non-bonding contacts and the loss in Accessible Surface Area (ASA).

Interacting residues	No. of hydrophobic contacts	ΔASA (Å^2^)
Ser-31	2	25.89^5^
Asp-32	12	57.88^2^
Asn-53	2	61.70^1^
Ser-56	2	17.52^6^
Leu-110	4	39.73^4^
His-196	2	47.72^3^

The ranking of residues on the basis of loss in solvent accessibility is indicated by superscripts with the value of ΔASA.

**Table 4:** The human AKT3 residues interacting with MK-2206 are listed with the number of non-bonding contacts and the loss in Accessible Surface Area (ASA).

Interacting residues	No. of hydrophobic contacts	ΔASA (Å^2^)
Met-1	1	23.72^3^
Thr-31	1	16.91^6^
Leu-109	1	11.77^7^
His-192	2	23.41^5^
Thr-193	2	23.54^4^
Leu-194	2	29.20^2^
Arg-198 (H-bond)	2	33.25^1^

The ranking of residues on the basis of loss in solvent accessibility is indicated by superscripts with the value of ΔASA. The residue forming hydrogen-bond is indicated with the residue name in parentheses.

**Table 5:** The binding strength of MK-2206 to the three AKT isoforms given by various scores are listed in the table.

AKT isoform	Binding energy	pK_d_ or −log(K_d_)	Dock score
AKT1	−8.83	6.47	−26.55
AKT2	−8.29	6.07	−37.05
AKT3	−7.48	5.48	−25.00

### Comparison between binding mode of MK-2206 and AKT inhibitor, inhibitor VIII

**Figure 6:**
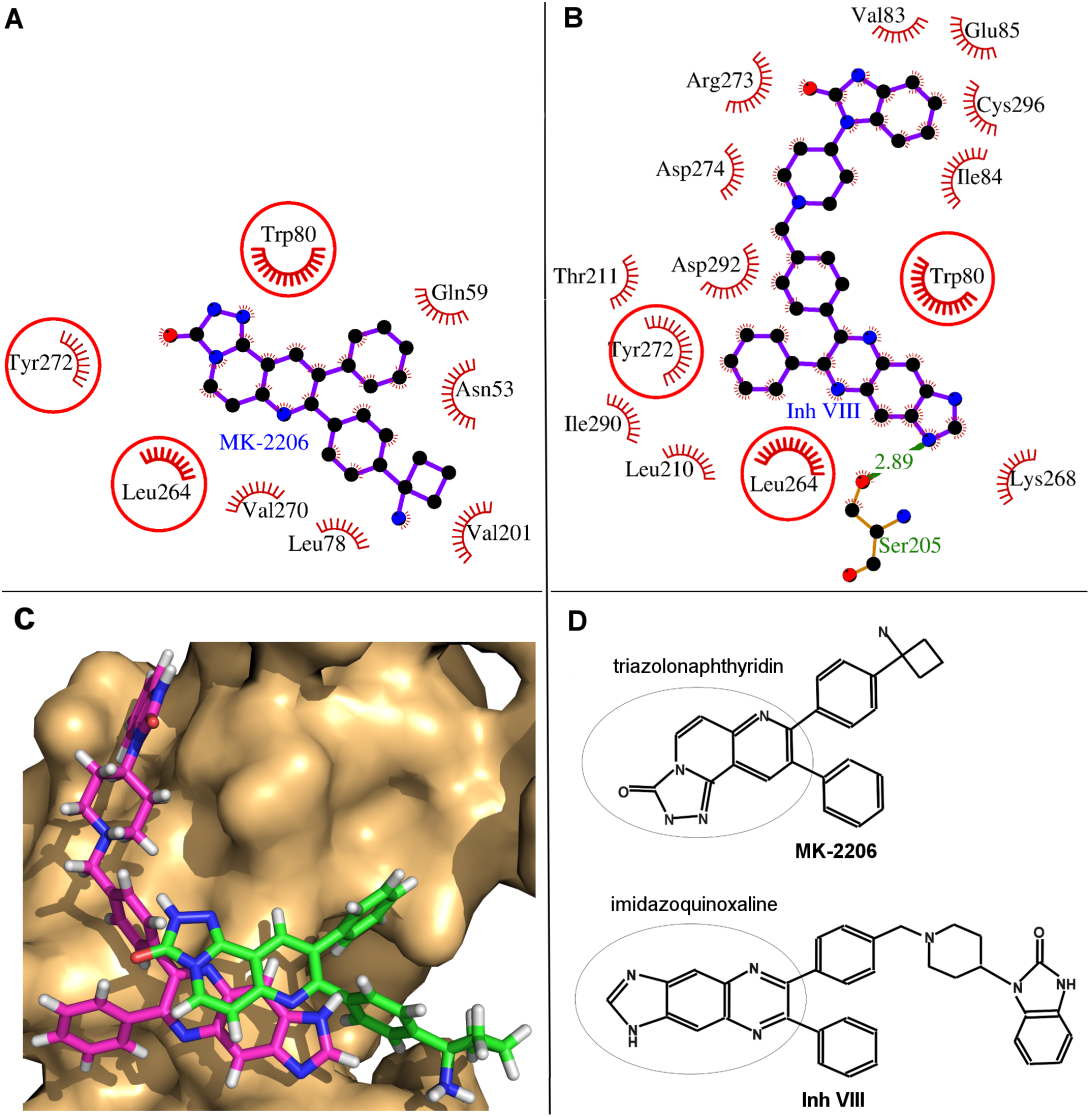
Comparative binding analyses of MK-2206 and inhibitor VIII. Panels A–B: The binding of MK-2206 and inhibitor VIII are displayed. The hydrogen bond is shown as green-dashed line with indicated bond length and the residues involved in hydrophobic interactions are shown as red arcs. The interacting residues which are common for both the ligands are encircled. Panel C: The exact orientation of binding for both the ligands in the binding site of the protein is shown. Panel D: Schematic structure of MK-2206 and inhibitor VIII are shown. The three ring moieties of both the molecules are encircled.

The human AKT1 structure chosen for docking analyses (PDB ID: 3O96) is available in PDB as co-complex structure crystallized with an allosteric inhibitor, inhibitor VIII [13]. In order to determine the difference between the binding mode of MK-2206 to AKT1 from that of inhibitor VIII, a comparative analyses was performed. It was found that the common interacting residues for both the ligands were Trp-80, Tyr-272 and Leu-264 (Fig. 6A–B). With respect to these common three interacting residues, inhibitor VIII binding site is towards the region encompassing residues Ile-84, Glu-85, Val-183, Thr-211, Arg-273, Asp-274, Asp-292, and Cys-296 whereas MK-2206 binding site is in opposite direction, the region involving the residues Asn-53, Glu-59, Leu-78, Val-201, and Val-270 (Fig. 6A–B). Alternatively, with respect to the terminal three-ring moiety of the ligands localized at the same small region in the allosteric site, their orientations are in opposite direction to each other (Fig. 6C–D).

### (Un)binding simulation analyses of MK-2206

The docked complex of AKT1 with MK-2206 was subjected to (un)binding simulation using MoMA-LigPath. The (un)binding simulation analyses of MK-2206 binding provided snapshots of varying molecular interactions with respect to decreasing distance from the binding site (Fig. 2A–I). While describing the (un)binding simulation analyses, we introduced two terminologies, ‘Common residues’ and ‘Additional residues’ to describe the two kinds of residues playing role in different phases of (un)binding simulation. The Common residues are the residues which are overlapping with the identified interacting residues of AKT1 (Table 1) whereas, the Additional residues are the residues which play role in binding at a certain phase of (un)binding simulation but they are not part of listed interacting residues in Table 1. The (un)binding simulation analyses of MK-2206 is briefly summarized as follows: In the first phase, Phase A (Fig. 2A), in addition to a Common residue, Gln-59, which is one of the identified listed interacting residues, there were Additional residues viz Ser-216, Asn-199, Leu-202, and Lys-111 that played role in initial binding of the ligand to the surface of the protein. In Phase B (Fig. 2B), another residue, Gln-218, was also involved as an Additional residue besides the Common residues, Trp-80 and Val-201, that interacted with the ligand. In Phase C (Fig. 2C), the ligand was bound by both the hydrogen bonds and hydrophobic interactions. The hydrogen bonds were formed by two Additional residues Asn-199 and Ser-205, whereas, the hydrophobic interactions were exerted by both Common residues, Trp-80 and Val-201, and Additional residues, Lys-111, Leu-202, Ala-58, and Leu-110. In Phase D (Fig. 2D), the Additional residues Leu-110, Leu-202 and, those forming hydrogen bonds (Asn-199, Ser-205) and one Common residue Val-201 disappeared. Whereas two Additional residue (Ser-56, Lys-268) and two Common residues (Asn-53, Glu-59) appeared forming interactions. It can be observed as the ligand is approaching towards the binding site, the number of Common residues increases with the decrease in the number of Additional residues. In the Phase E (Fig. 2E), an Additional residue, Lys-268, which was present in Phase D also, formed a hydrogen-bond with the ligand. The Common residue Gln-59 is replaced by Val-270 and the Additional residue Lys-111 is replaced by Ser-205. However, the number of Additional and Common residues remained the same at this phase similar to Phase D. In Phase F (Fig. 2F), the hydrogen bond disappeared but the Additional residue forming this hydrogen bond, Lys-268 was still there forming hydrophobic interactions in this phase. Other residues remained same except the Additional residue Ala-58 which disappeared at this phase. In Phase G (Fig. 2G), one Common residue Glu-59 appeared with the disappearance of one Additional residue Ser-56. In Phase H (Fig. 2H), the Common residues appeared were Leu-78, Val-201, and, Leu-264 with the disappearance of one Common residue Glu-59. In Additional residues, Gln-79 appeared with the disappearance of Ser-205. The number of Additional residues remained same but the number of Common residues increased. Finally, in the binding site phase, Phase I (Fig. 2I), all the Additional residues disappeared. In the Common residues, Gln-59 which was present at Phase G reappeared and another Common residue Tyr-272 appeared completing the quorum of the Common residues.

### Molecular docking study of structural analogs of MK-2206 to AKT1

**Figure 7:**
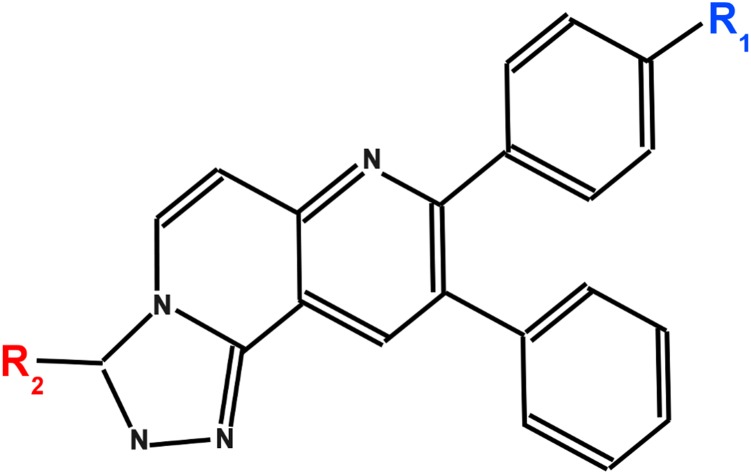
Structural analogs of MK-2206 can be derived by varying R1 and R2 group on common scaffold according to devised rules on visual analyses. For the drug MK-2206, R_1_ is 1-amino cyclo-butyl group and R_2_ is ketonic oxygen.

The careful analyzes of all the 30 structural analogs for common scaffold led us to devise rules to classify the compounds in three groups. We found that the compounds are derivatives of common scaffold with varying R_1_ and R_2_ group as shown in Fig. 7. These compounds were, therefore, classified in three groups as R_1_-, R_2_- and R_1_R_2_- structural analogs of MK-2206 depending on the substitutions made at R_1_- or R_2_- or both on the common scaffold. The docking of all these structural analogs to the allosteric site of AKT1 was carried out. The ligand-interaction plots of all the 30 structures grouped according to R_1_-, R_2_- and R_1_R_2_- classes are provided as Files S1–S3. Except for one belonging to R_1_-class which has no overlapping interacting residues with MK-2206 i.e. binding to different site, all the structure analogs have Trp-80 as common interacting residues. This may gives an idea about the binding of the common scaffold and it also underscores the importance of Trp-80 in the scaffold binding. In case of R_1_-class, despite Trp-80, the other residue Asn-53 was also common. Whereas, in R_2_-class, in addition to Trp-80 and Asn-53 which were common in R_1_-class, another residue Val-270 was also found common. Finally, in R_1_R_2_-class, Trp-80 was the only common residue whereas Asn-53 and Val-270 including Tyr-272 were frequently appearing as common residues among many structural analogs. The dock score is directly obtained from Dock v.6.5 [28], whereas binding energy and pK_d_ (for dissociation constant) were calculated using X-Score v.1.2.11 [35]–[36]. All these scores for all the structural analogs are tabulated in Table S1. With few exceptions, the dock scores are not varying much with respect to that of MK-2206. In order to identify the better inhibitor than the MK-2206, we found a compound with CID 67256123 as the best binder with highest binding energy and pK_d_ among the structural analogs of MK-2206. We believe this R_2_-structural analog can prove to be better inhibitor than MK-2206 provided it qualifies the low toxicity and better oral-availability criteria in vivo.

### Comparison between binding mode of MK-2206 and a proposed better inhibitor, an MK-2206 analog

**Figure 8:**
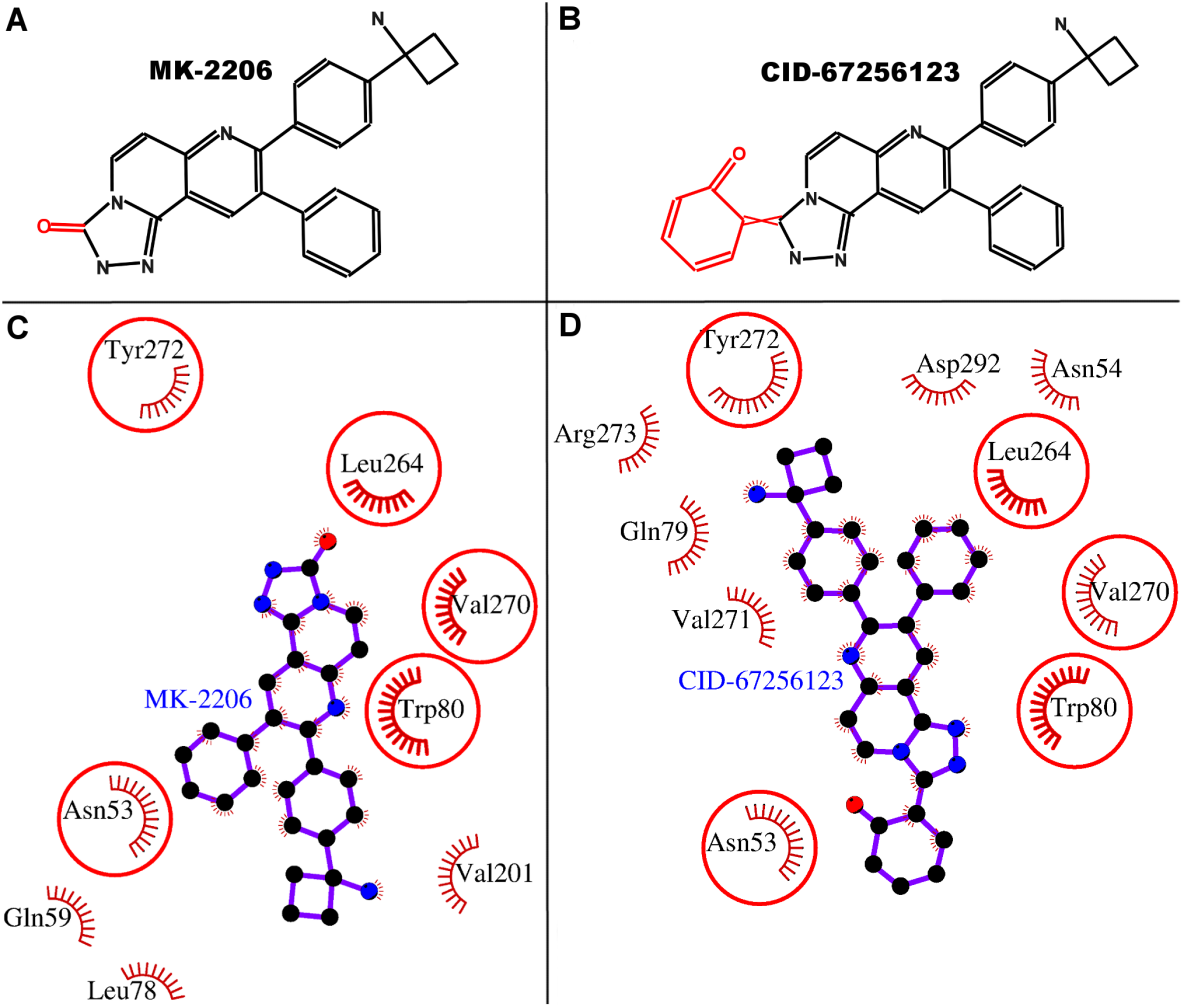
Comparative binding analyses of MK-2206 and its selected analog. Panels A–B: Schematic structure of MK-2206 and its analog are shown. The only difference between the compounds is change in R_2_ group, shown in red on black compound-scaffold. Panel C–D: The binding of MK-2206 and its selected analog are displayed. The residues involved in hydrophobic interactions are shown as red arcs. The interacting residues which are common for both the ligands are encircled.

This R_2_-structural analog of MK-2206 is having cyclohexa-2,4-dien-1-one group on R_2_ instead of mere ketonic oxygen in MK-2206 (Fig. 8A–B). The common interacting residues between the two ligands were Asn-53, Trp-80, Leu-264, Val-270, and Tyr-272 (Fig. 8C–D). The two ligands bind in the same location within the allosteric site, however, the orientations of the analogs were inverted. In case of MK-2206, the two phenyl groups protruding from three ring-structure triazolonaphthyridin moiety face towards the surface of the protein within the allosteric site and not involved in molecular interactions with many residues. Whereas, in case of the MK-2206 analog, the two protruding phenyl groups from triazolonaphthyridin moiety face towards deep inside the cavity and involved in multiple interactions and thus provide better fit than the original drug MK-2206. This is also evident from the total number of molecular interactions in MK-2206 analog (40 interactions out of 10 residues, Table 6) which drastically increased from that of MK-2206 (25 interactions out of 8 residues, Table 1). Although the dock scores of both the drugs were similar but the binding energy of MK-2206 analog was higher than that of MK-2206 and the dissociation constant of analog was approximately 10 times of the drug MK-2206. All these findings suggest that the MK-2206 analog is proposed to be a better inhibitor than the original drug MK-2206.

**Table 6:** The AKT1 residues interacting with MK-2206 analog are listed with the number of non-bonding contacts and the loss in Accessible Surface Area (ASA).

Interacting residues	No. of hydrophobic contacts	ΔASA (Å^2^)
Asn-53	6	51.38^2^
Asn-54	2	13.83^9^
Gln-79	9	37.29^4^
Trp-80	7	66.43^1^
Leu-264	1	14.21^8^
Val-270	5	37.86^3^
Val-271	2	6.11^10^
Tyr-272	4	36.18^5^
Arg-273	1	31.28^6^
Asp-292	3	15.67^7^

The ranking of residues on the basis of loss in solvent accessibility is indicated by superscripts with the value of ΔASA.

## Conclusions

The present study used docking and (un)binding simulation analyses to identify MK-2206 interacting residues of human AKT isoforms. The MK-2206 is an allosteric inhibitor of AKT1 and exerts its inhibitory mechanism by binding to the allosteric site of AKT1 and engaging the functionally important residues in various interactions. The exact binding mode of MK-2206 based on computational approach is presented and various interacting residues within the allosteric site of this protein were identified and characterized. The quality of docking was assured by the negative dock score with high absolute value and the identified various molecular interactions between the protein and the ligand. Additionally, the extent of involvement of the residues in ligand binding was calculated by ASA analyses and the residues were ranked on the basis of ΔASA score. In the docking and (un)binding simulation analyses, the Trp-80 was the key residue among various important identified residues, and showed highest decrease in the solvent accessibility, highest number of hydrophobic interactions, and the most consistent involvement in all (un)binding simulation phases. The AKT1 residues interacting with MK-2206 were also compared with those of other AKT isoforms. The lowered binding affinity of AKT3 to MK-2206 is attributed to decreased number of molecular interactions and lowered calculated- binding energy and dissociation constants. The (un)binding simulation analyses identified various Additional residues which despite being away from the binding site play important role in initial binding of the ligand and its recruitment to the binding site of the AKT1 protein. The molecular docking analyses of MK-2206 structural analogs identified one structural analog proposed as better inhibitor of AKT1 than MK-2206. Thus, the aforementioned docking and (un)binding analyses provide the structural insights into the binding mechanism of MK-2206 to the isoforms of key cancer signaling protein, AKT. The docked MK-2206–protein conformation is expected to serve as a suitable model for understanding the drug protein interplay or more specifically the amino-acid environment mediating molecular-interactions and thus, providing electrostatic and surface complementary details for the inhibitory mechanism.

## Supporting Information

File S1 **R1-analogs of MK-2206.**
(PDF)

File S2 **R2-analogs of MK-2206.**
(PDF)

File S3 **R1R2-analogs of MK-2206.**
(PDF)

Table S1 **The binding strength of structural analogs of MK-2206 to human AKT1 given by various scores are listed in the table.** These are classified as R1-, R2- and R1R2- structural analogs of MK-2206 as described in Materials and Method section.
(DOC)
