# Supplementary material for: Computational Insights into the Inhibitory Mechanism of Human AKT1 by an Orally Active Inhibitor, MK-2206
Source: PLoS One. 2014 Oct 17;9(10):e109705. doi: 10.1371/journal.pone.0109705 (PMC4201482; doi:10.1371/journal.pone.0109705)
Supplement: File S1 — R1-analogs of MK-2206. (PDF) [file pone.0109705.s001.pdf]

R_1-analogs of MK-2206

Ligand-interaction plots of R_1-analogs of MK-2206 are shown. The MK-2206 analogs' names are indicated by PubChem ID. The hydrogen bonds are shown as green-dashed lines with indicated bond length and the residues involved in hydrophobic interactions are shown as red arcs. The residues which are common to the first compound, the drug MK-2206 are encircled.

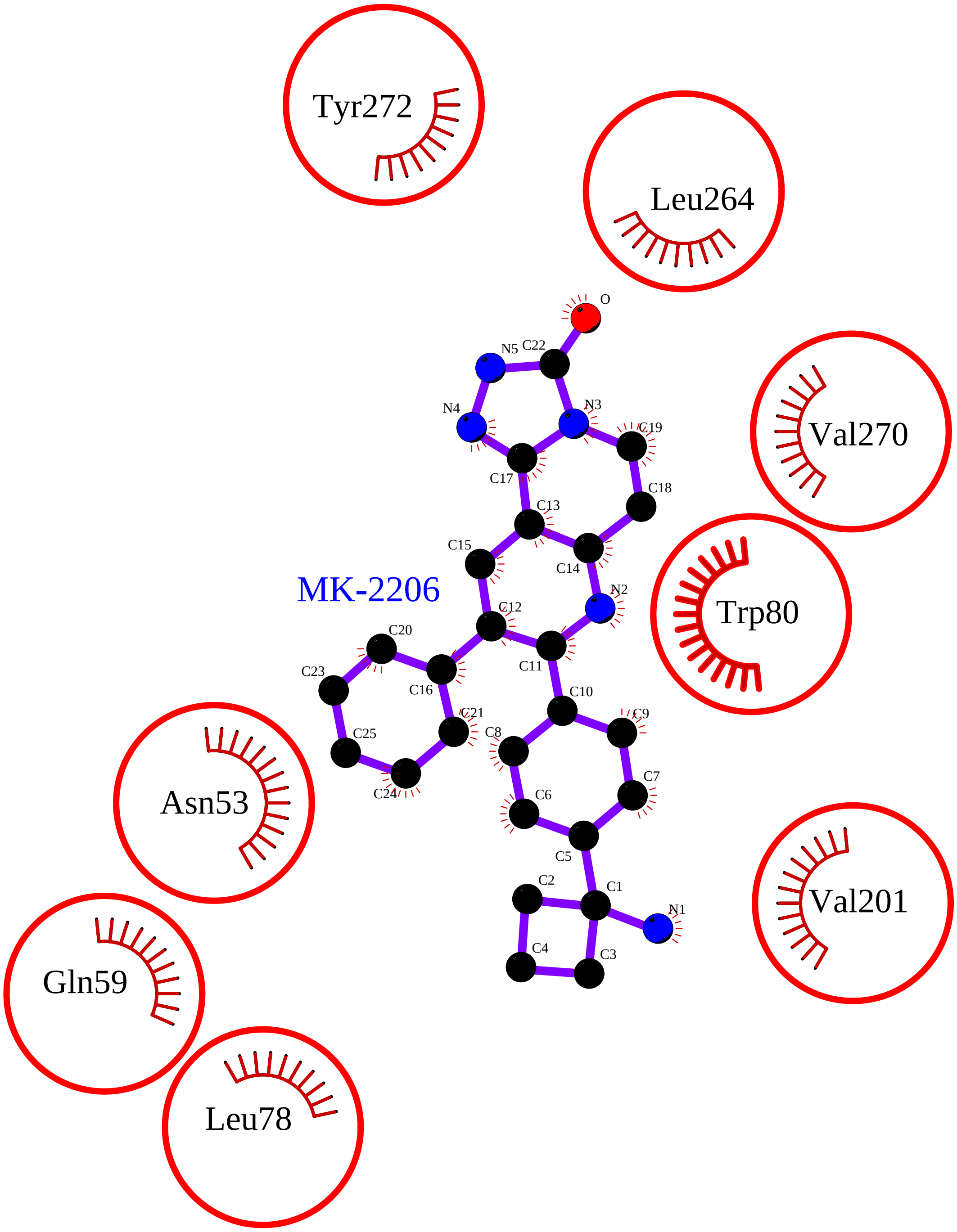

Tyr272
Leu264
O
N5
C22
N4
N3
C19
Val270
C17
C18
C13
C15
C14
MK-2206
N2
Trp80
C12
C20
C11
C23
C16
C10
C21
C9
C8
C25
Asn53
C7
C24
C6
C5
Val201
C2
C1
N1
C4
C3
Gln59
Leu78

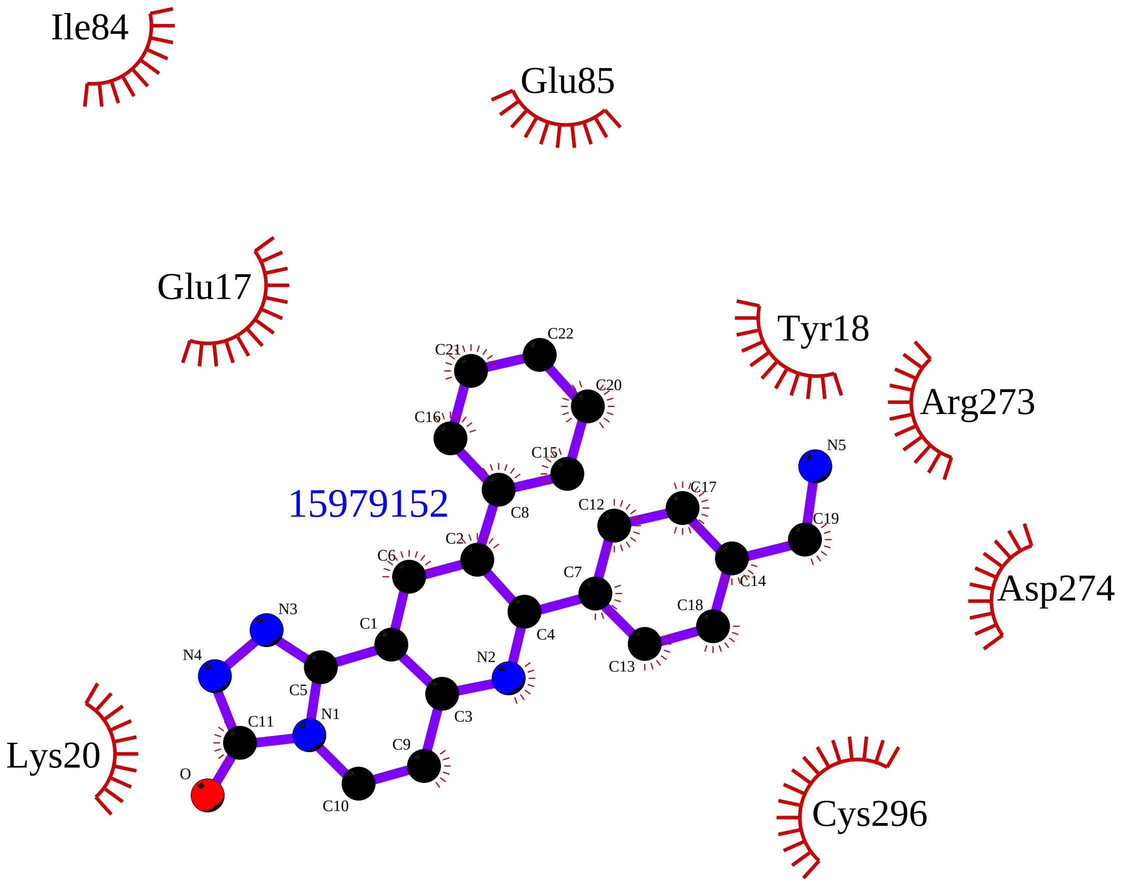
Ile84
Glu85
Glu17
Tyr18
Arg273
15979152
Asp274
Lys20
Cys296
C21
C22
C20
C16
C15
C8
C12
C17
N5
C19
C6
C2
C7
C14
C18
N3
C1
C4
N4
N2
C13
C5
C11
N1
C3
O
C9
C10

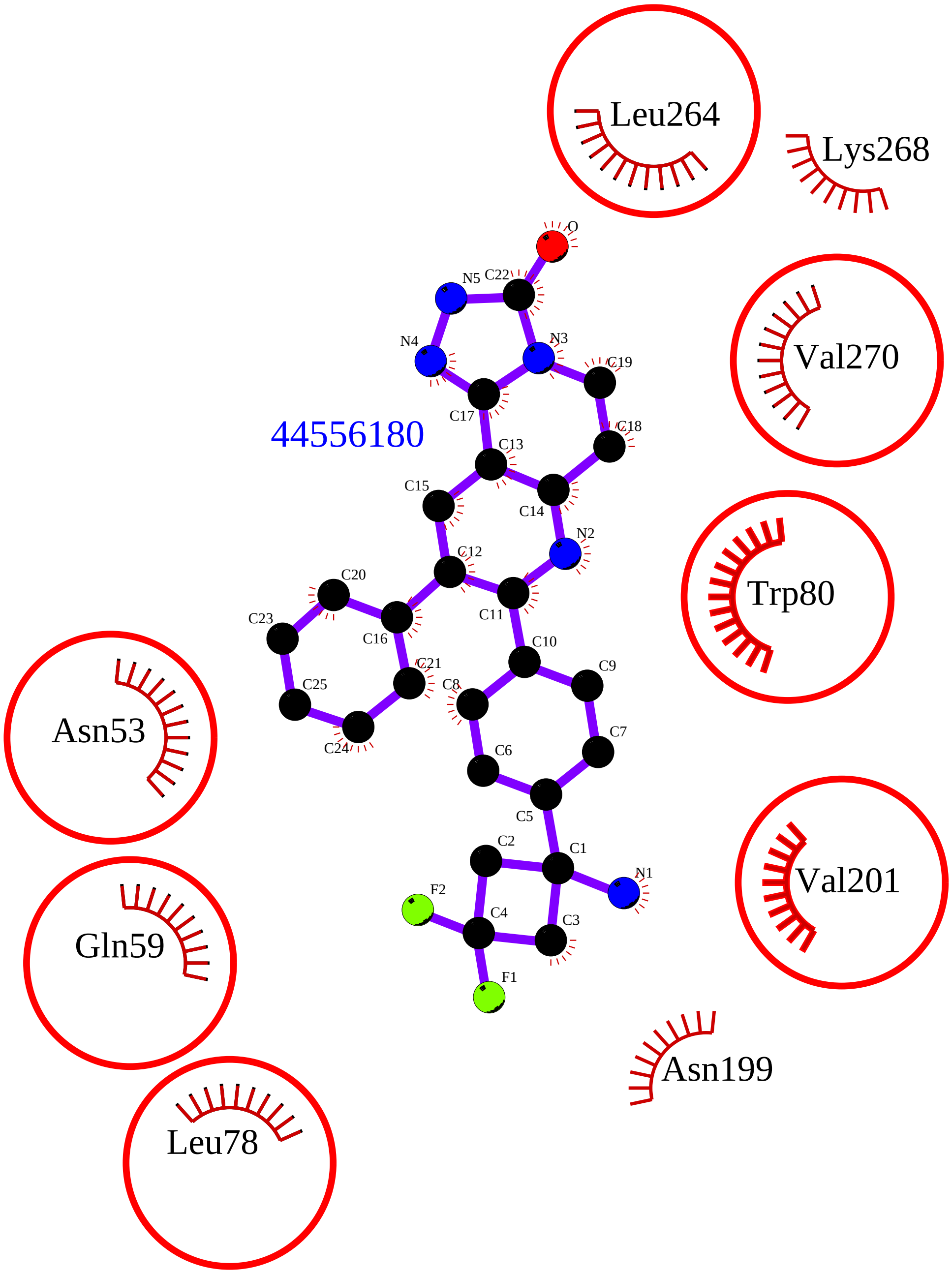

Leu264
Lys268
Val270
44556180
Trp80
Asn53
Val201
Gln59
Asn199
Leu78
O
N5
C22
N4
N3
C19
C17
C18
C13
C15
C14
N2
C12
C20
C11
C23
C16
C10
C21
C9
C25
C8
C24
C7
C6
C5
C2
C1
N1
F2
C4
C3
F1

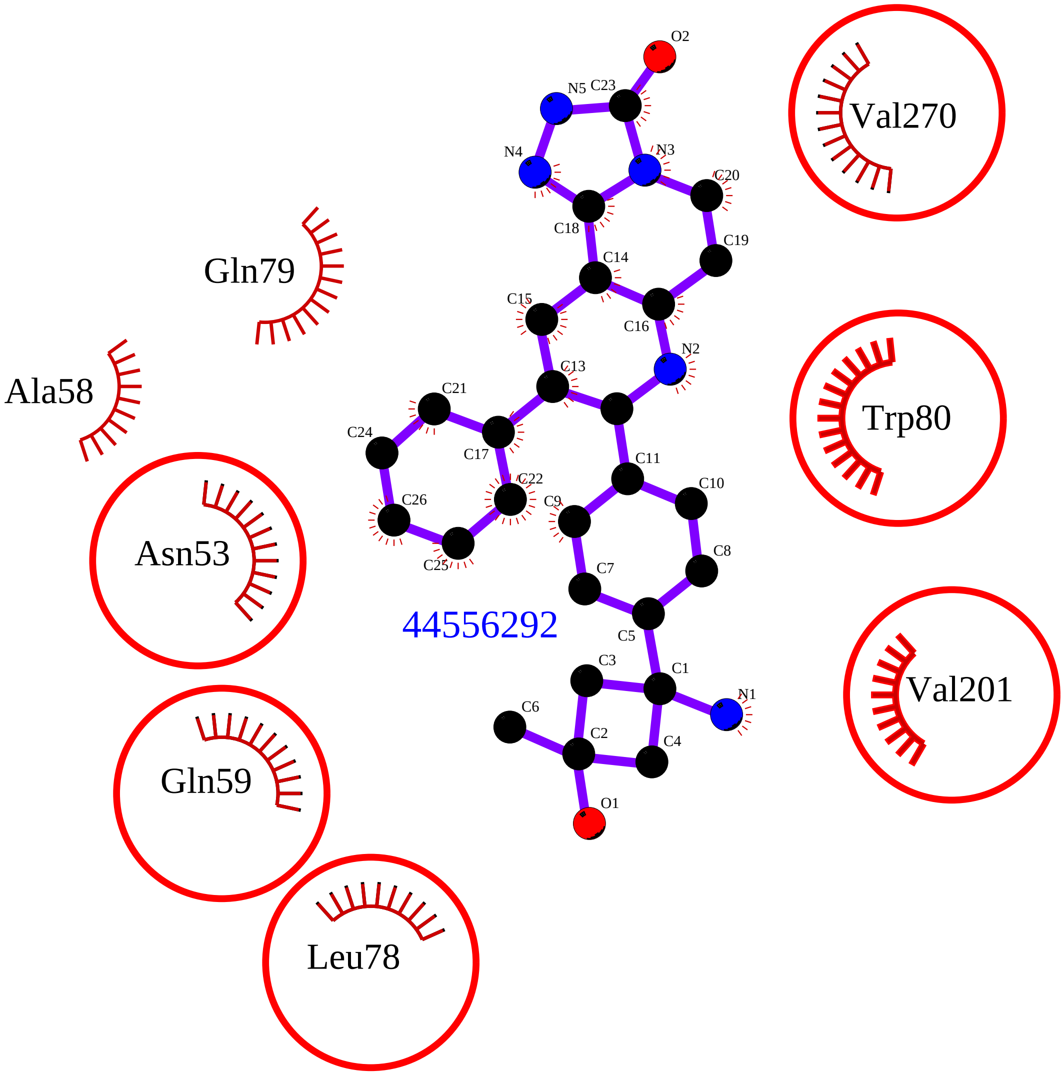

O2
N5
C23
N4
N3
C20
C18
C19
C14
C15
C16
N2
C13
C21
C24
C17
C22
C11
C10
C9
C26
C8
C25
C7
C5
C3
C1
N1
C6
C2
C4
O1
44556292
Val270
Gln79
Trp80
Ala58
Asn53
Val201
Gln59
Leu78

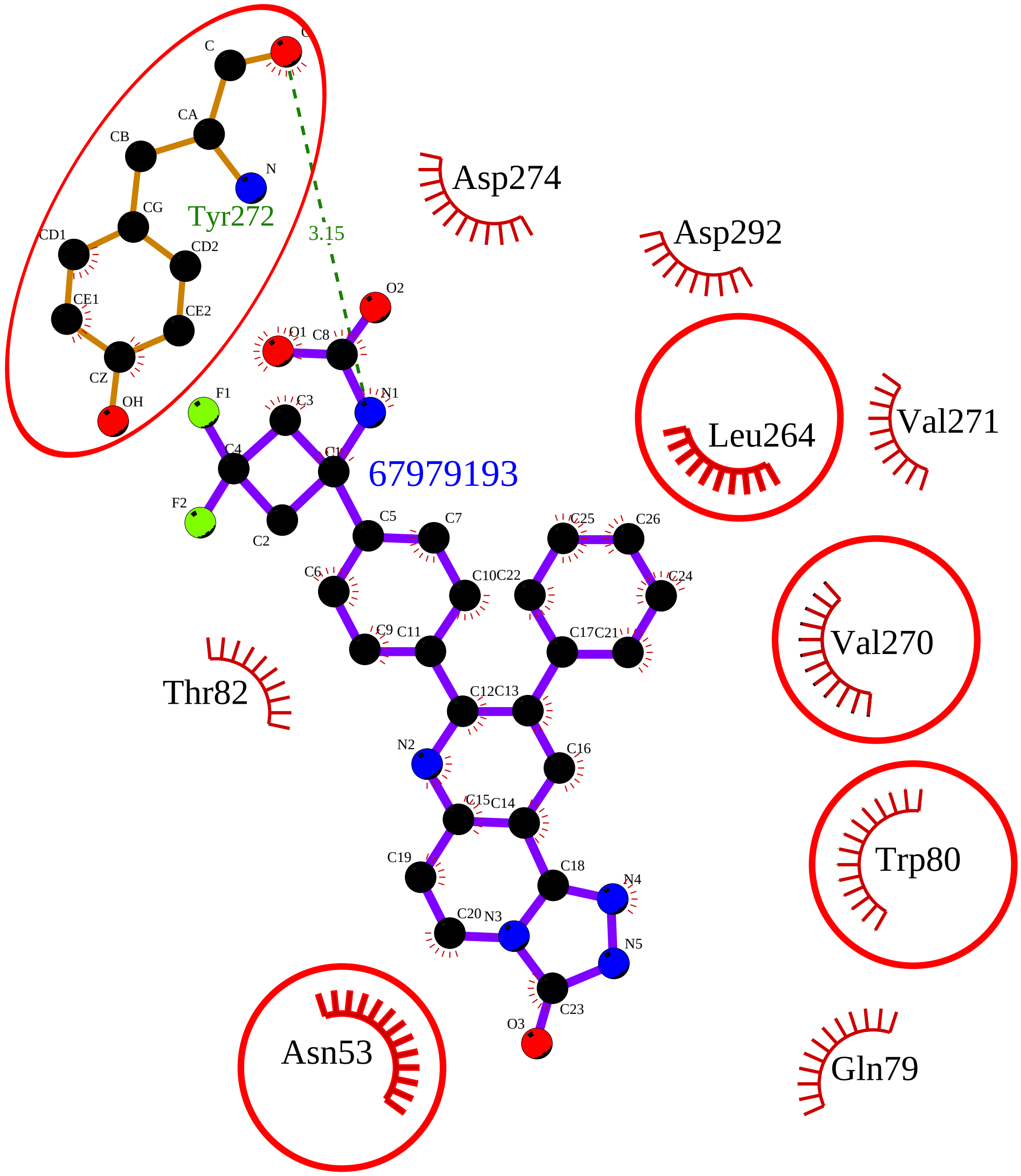

C
O
CA
CB
N
CG
Tyr272
CD1
CD2
3.15
CE1
CE2
O2
O1
C8
CZ
OH
F1
C3
N1
C4
C1
67979193
F2
C2
C5
C7
C6
C10
C22
C25
C26
C24
C9
C11
C17
C21
C12
C13
N2
C16
C15
C14
C19
C18
N4
C20
N3
N5
C23
O3
Asp274
Asp292
Leu264
Val271
Val270
Thr82
Trp80
Asn53
Gln79

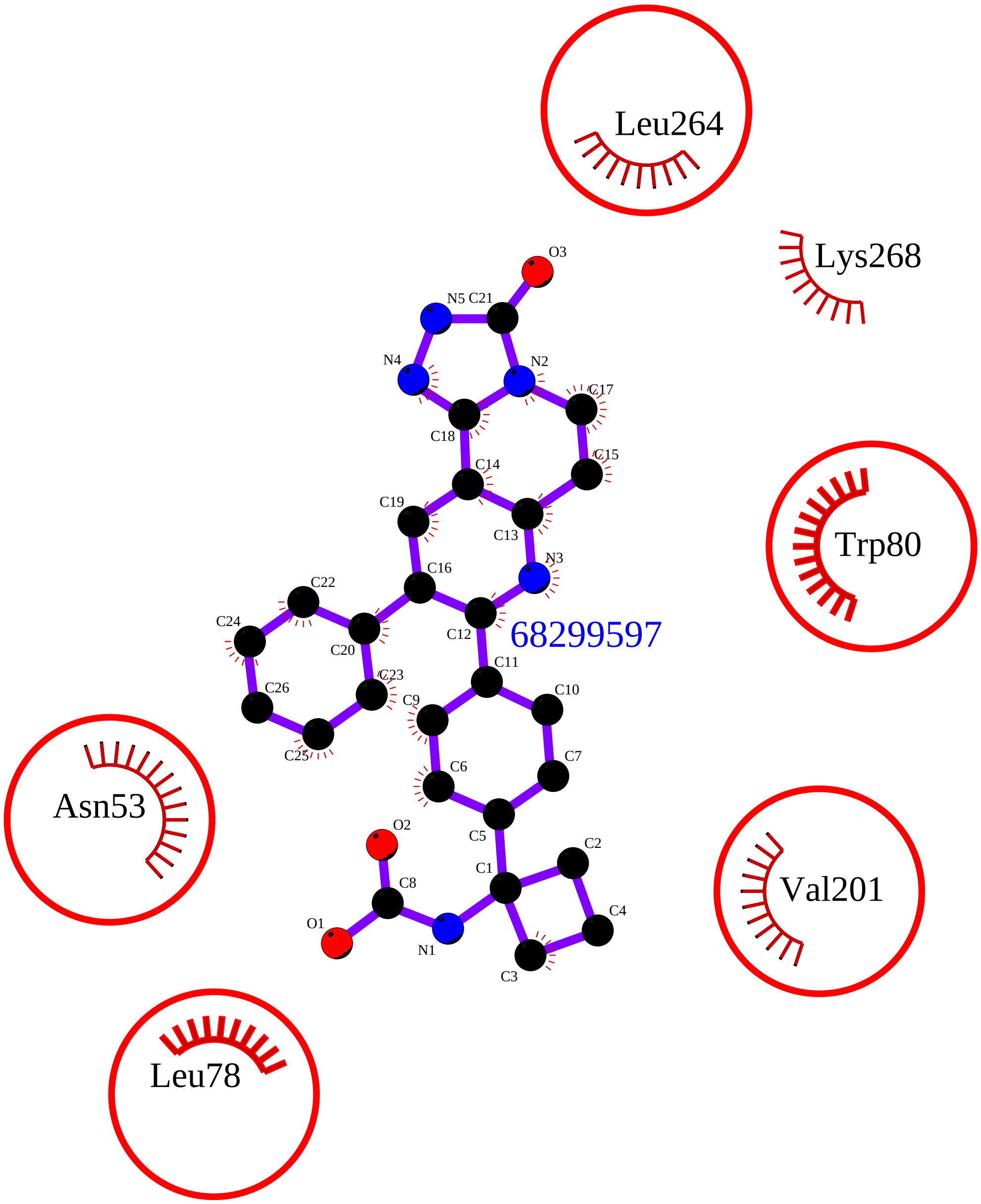

Leu264
Lys268
O3
N5 C21
N4
N2
C17
C18
C15
C14
C19
Trp80
C13
N3
C16
C22
C24
C20
C12
68299597
C11
C23
C10
C26
C9
C7
C25
C6
Asn53
C5
O2
C2
C1
Val201
C8
C4
O1
N1
C3
Leu78

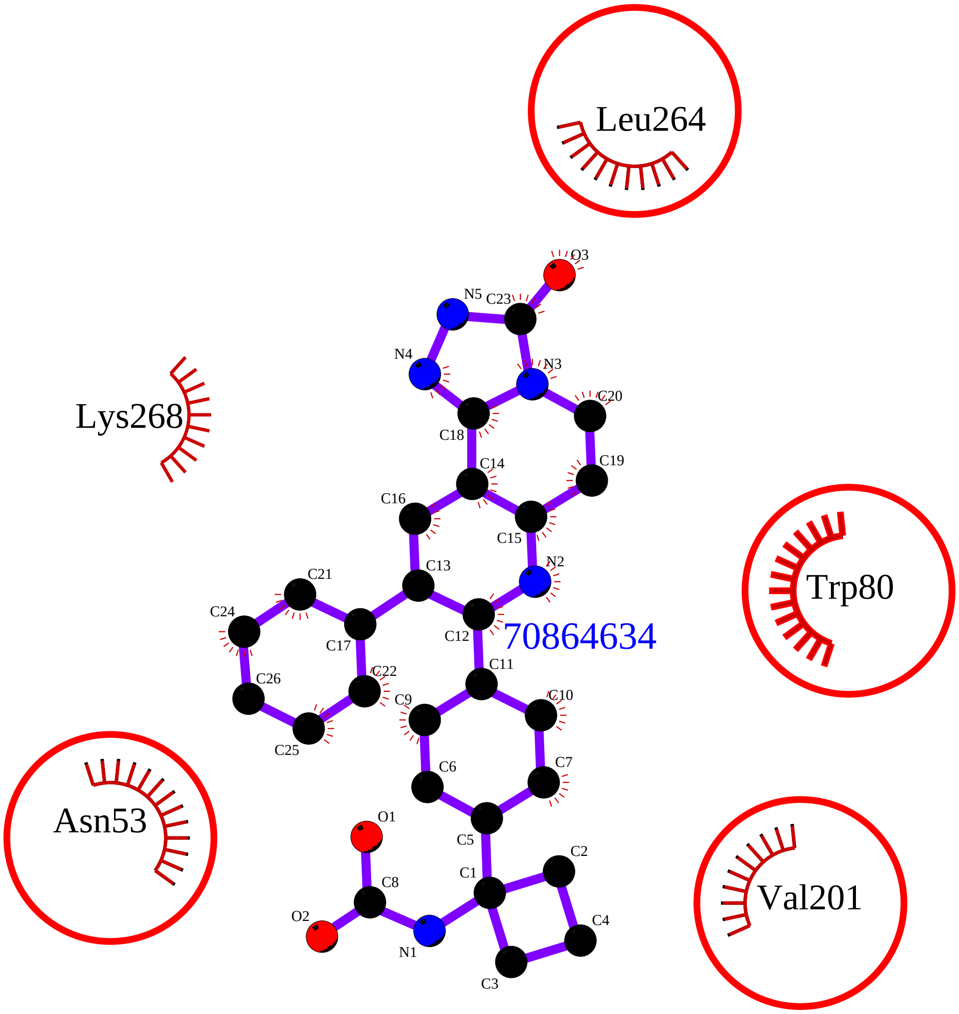

Leu264
O3
N5
C23
N4
N3
C20
Lys268
C18
C14
C19
C16
C15
Trp80
N2
C21
C13
C24
C17
C12
70864634
C11
C22
C26
C9
C10
C25
C6
C7
Asn53
O1
C5
C2
C8
C1
Val201
O2
N1
C4
C3
