# Supplementary material for: Computational Insights into the Inhibitory Mechanism of Human AKT1 by an Orally Active Inhibitor, MK-2206
Source: PLoS One. 2014 Oct 17;9(10):e109705. doi: 10.1371/journal.pone.0109705 (PMC4201482; doi:10.1371/journal.pone.0109705)
Supplement: File S2 — R2-analogs of MK-2206. (PDF) [file pone.0109705.s002.pdf]

R_2-analogs of MK-2206

Ligand-interaction plots of R_2-analogs of MK-2206 are shown. The MK-2206 analogs' names are indicated by PubChem ID. The hydrogen bonds are shown as green-dashed lines with indicated bond length and the residues involved in hydrophobic interactions are shown as red arcs. The residues which are common to the first compound, the drug MK-2206 are encircled.

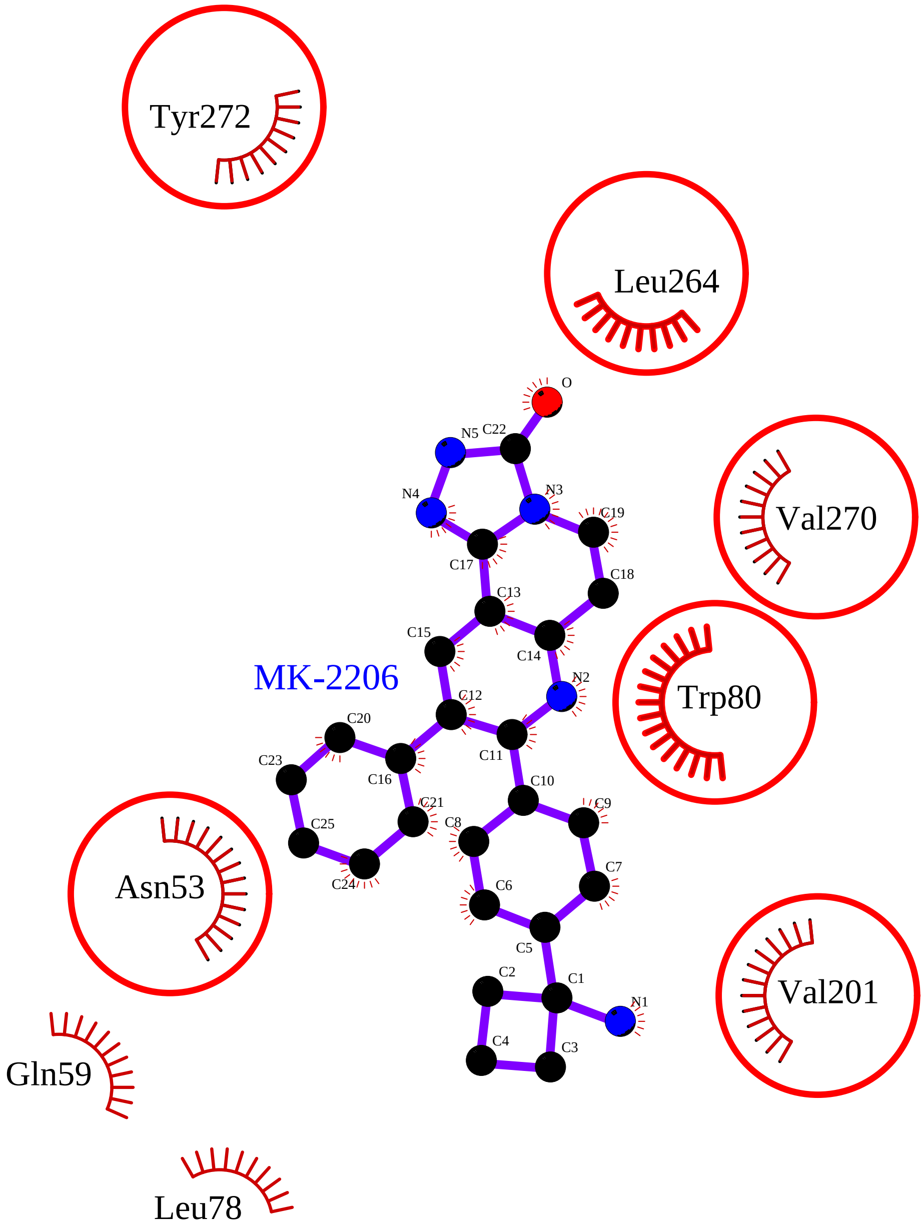
Tyr272
Leu264
Val270
Trp80
MK-2206
Asn53
Val201
Gln59
Leu78
O
N5
C22
N4
N3
C19
C17
C18
C13
C15
C14
N2
C12
C20
C11
C23
C16
C21
C10
C9
C8
C25
C24
C6
C7
C5
C2
C1
N1
C4
C3

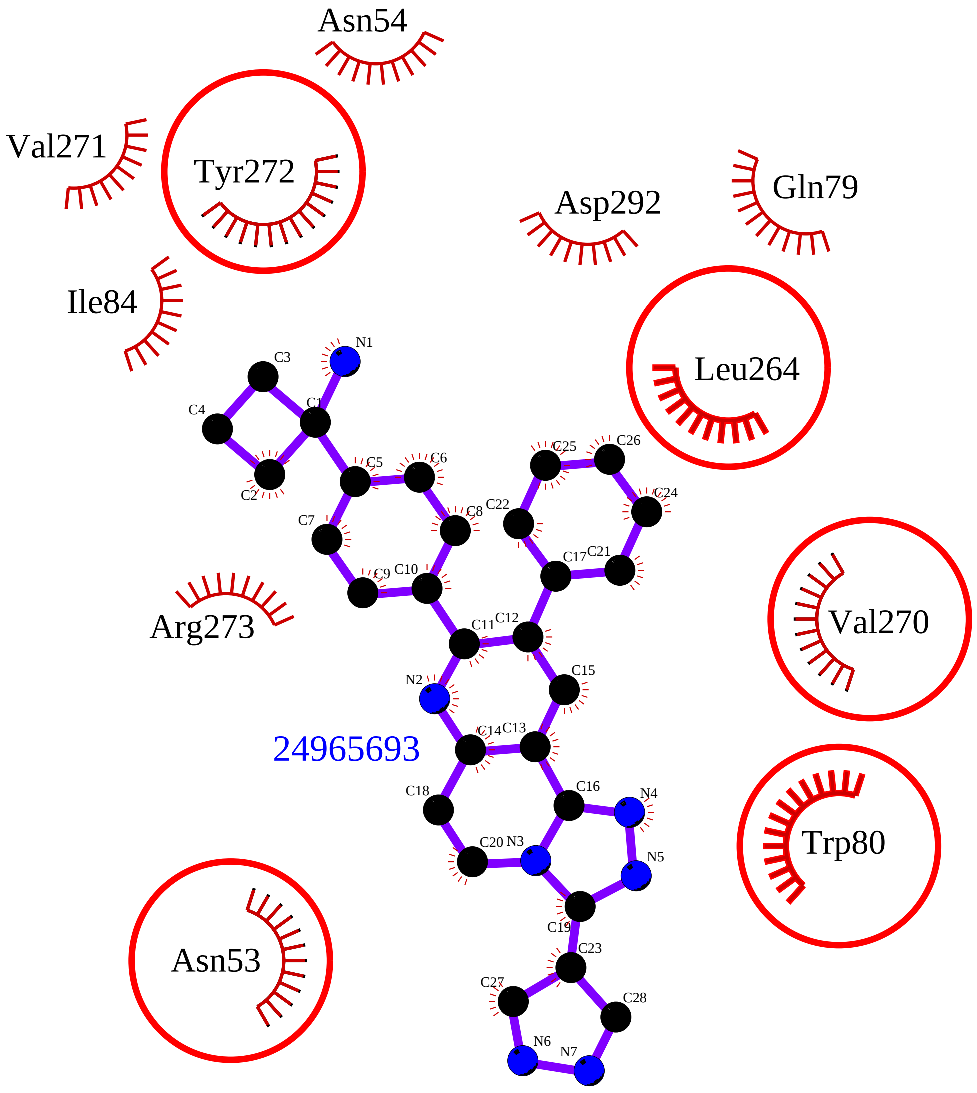

Asn54
Val271
Tyr272
Asp292
Gln79
Ile84
Leu264
Val270
Arg273
24965693
Trp80
Asn53
N1
C3
C4
C1
C2
C5
C6
C7
C8
C9
C10
C22
C25
C26
C24
C17
C21
C11
C12
N2
C15
C14
C13
C18
C16
N4
C20
N3
N5
C19
C23
C27
C28
N6
N7

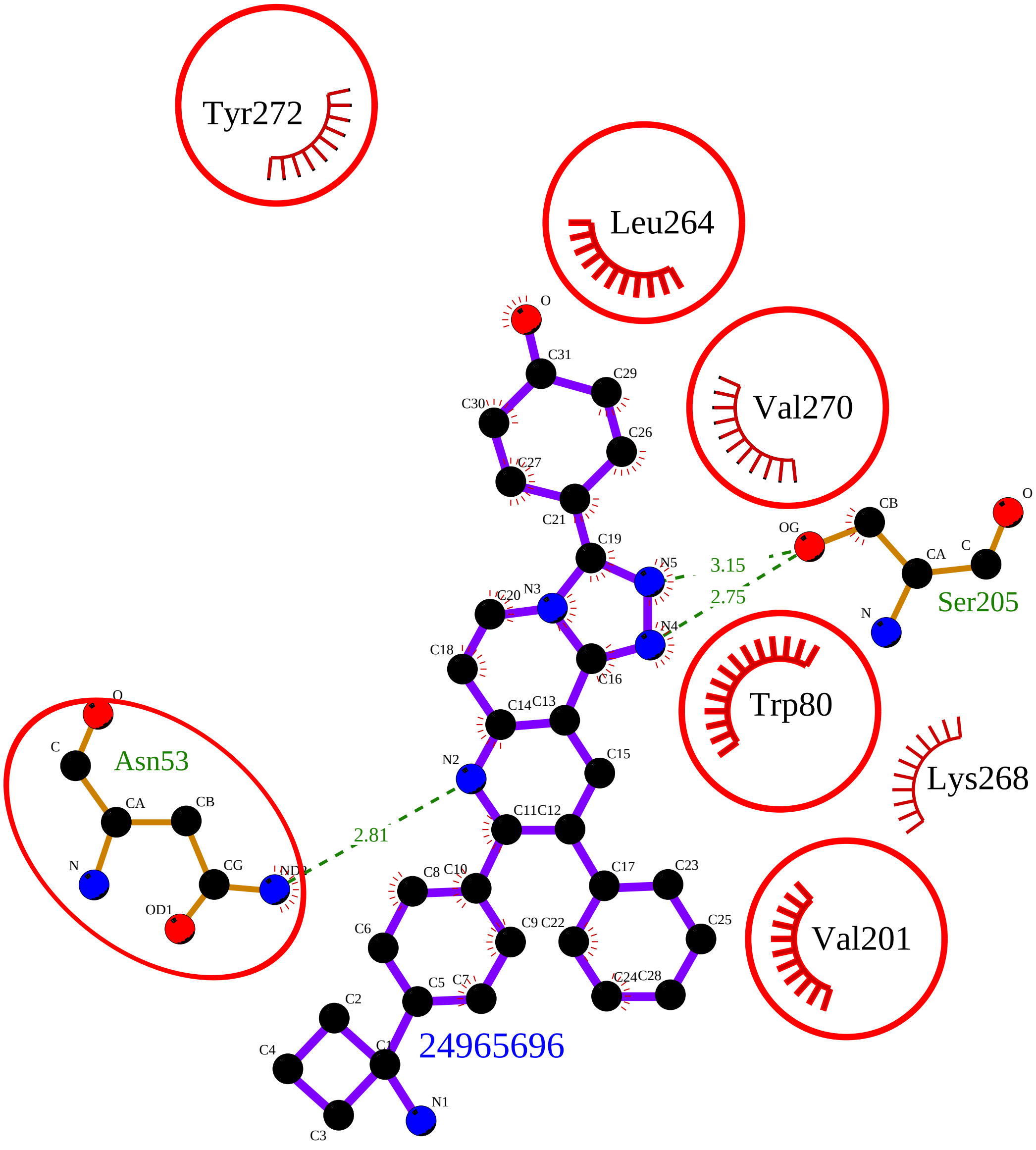

Tyr272
Leu264
Val270
Ser205
Trp80
Lys268
Asn53
Val201
24965696
3.15
2.75
2.81

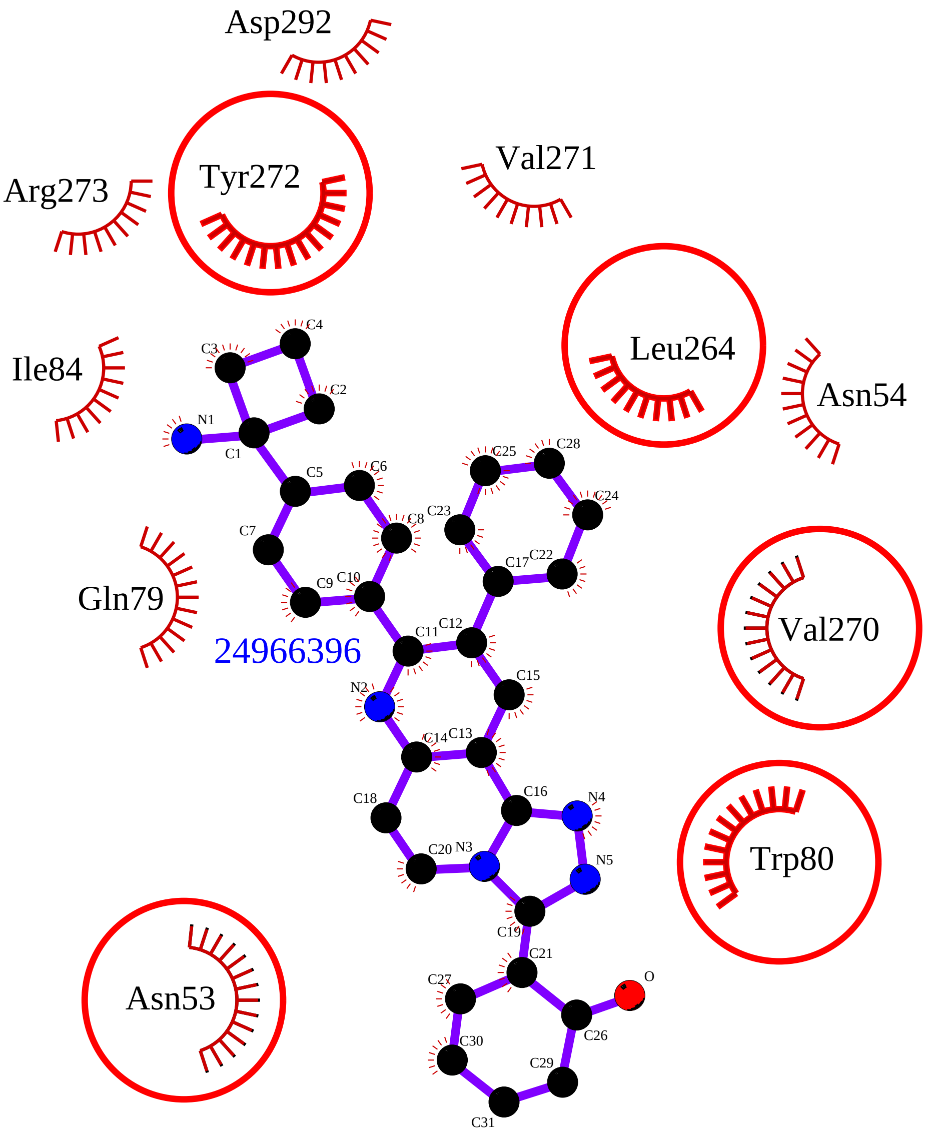
Asp292
Tyr272
Val271
Arg273
Leu264
Asn54
Ile84
Gln79
24966396
Val270
Trp80
Asn53

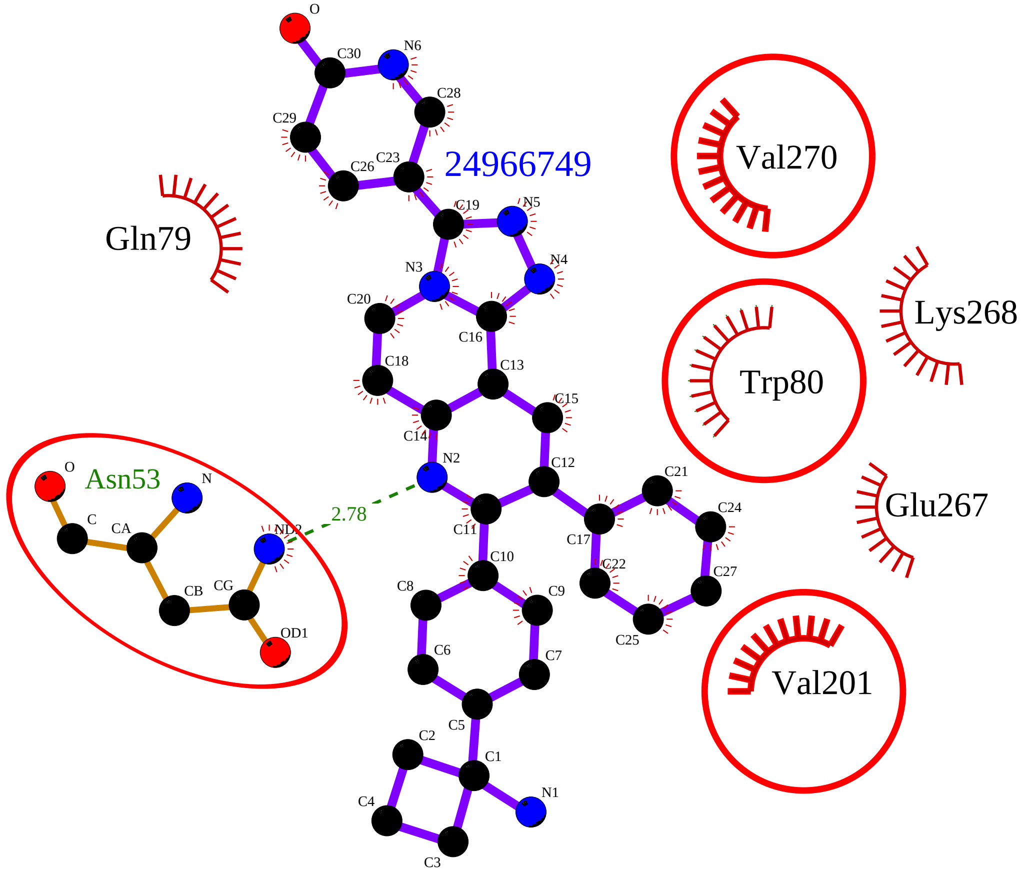

24966749
Gln79
Val270
Lys268
Trp80
Glu267
Val201
Asn53
2.78

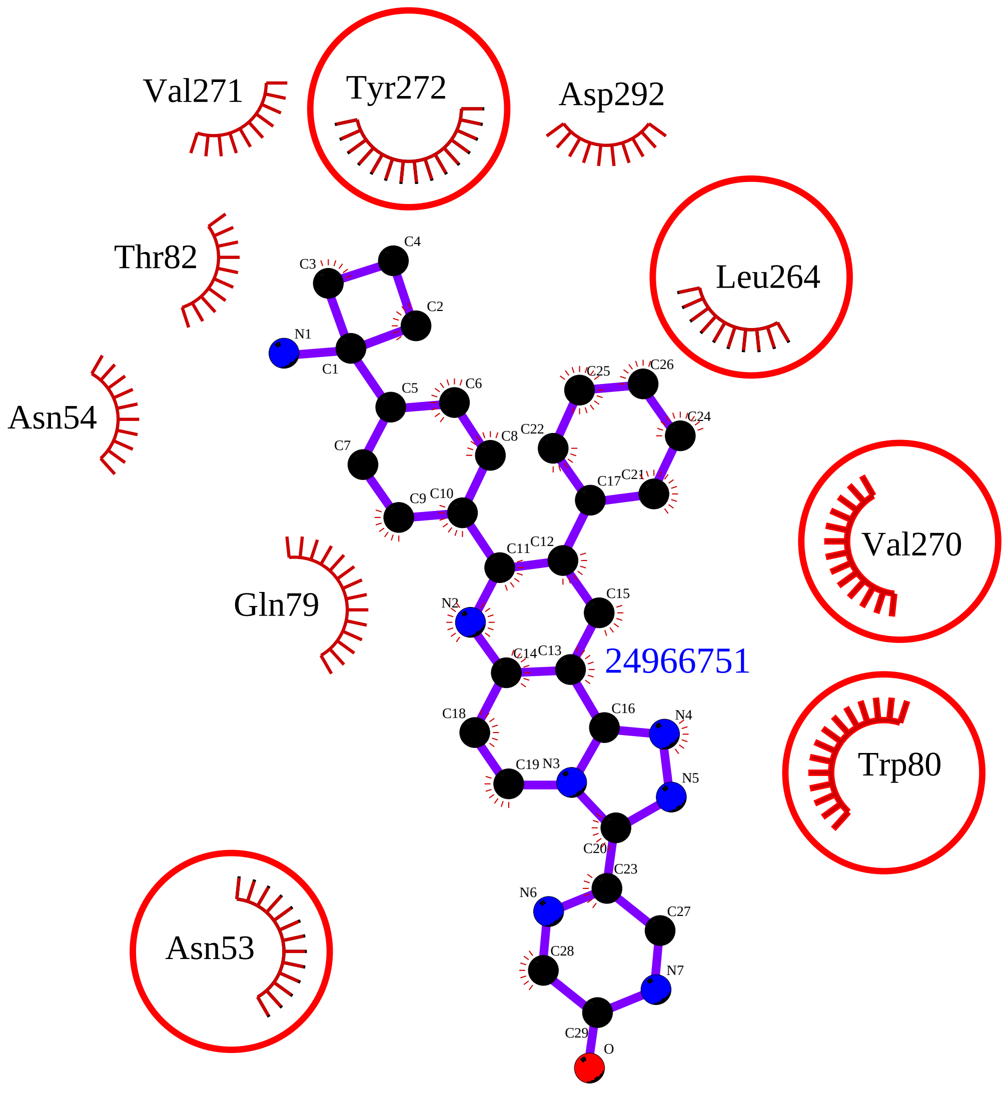

Val271
Tyr272
Asp292
Thr82
Leu264
Asn54
Val270
Gln79
24966751
Trp80
Asn53
N1
C1
C2
C3
C4
C5
C6
C7
C8
C9
C10
C11
C12
C13
C14
C15
C16
C17
C18
C19
C20
C21
C22
C23
C24
C25
C26
C27
C28
C29
N2
N3
N4
N5
N6
N7
O

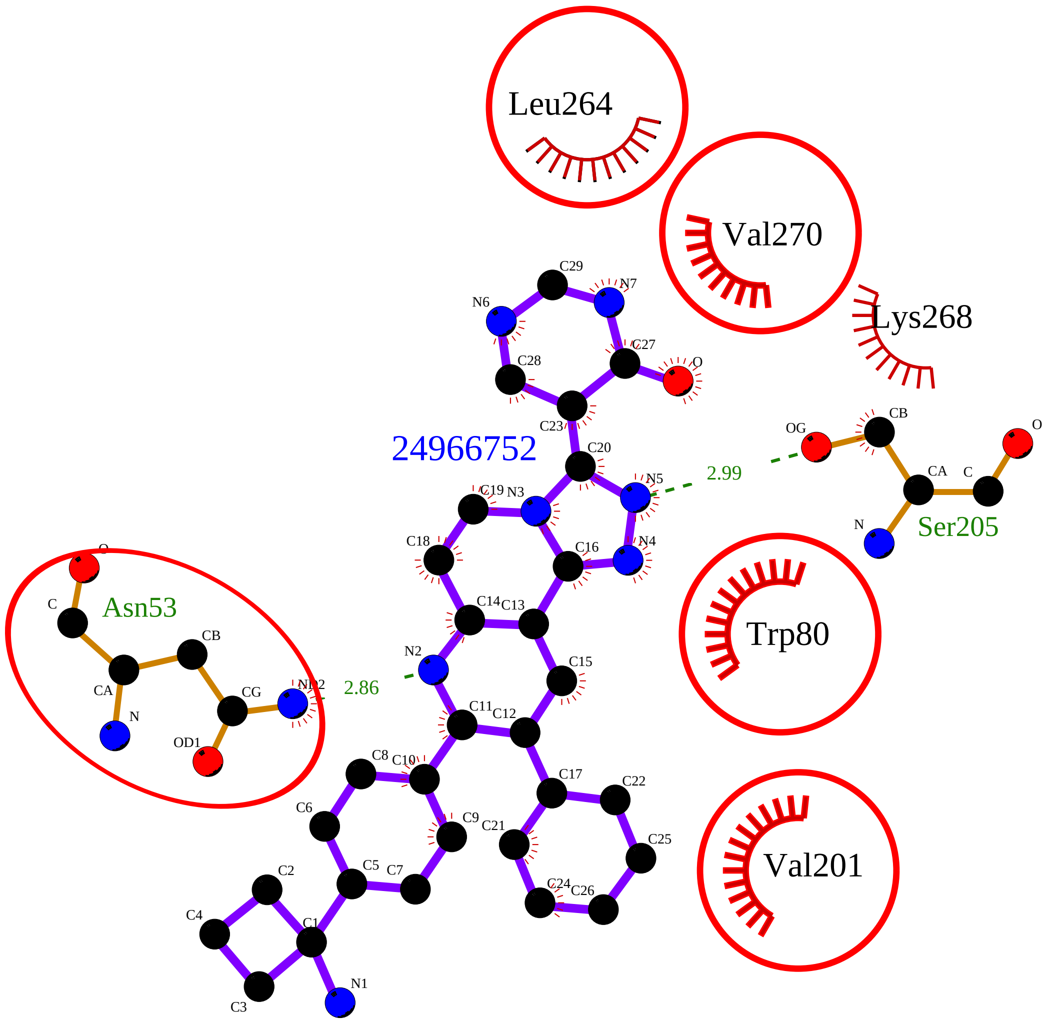

Leu264
Val270
Lys268
C29
N7
N6
C27
O
C28
C23
24966752
C20
OG
CB
O
N5
2.99
CA
C
C19
N3
N
Ser205
C18
C16
N4
O
Asn53
C
C14
C13
Trp80
CB
N2
C15
CA
CG
ND2
2.86
N
C11
C12
OD1
C8
C10
C17
C22
C6
C9
C21
C25
Val201
C5
C7
C2
C24
C26
C4
C1
C3
N1

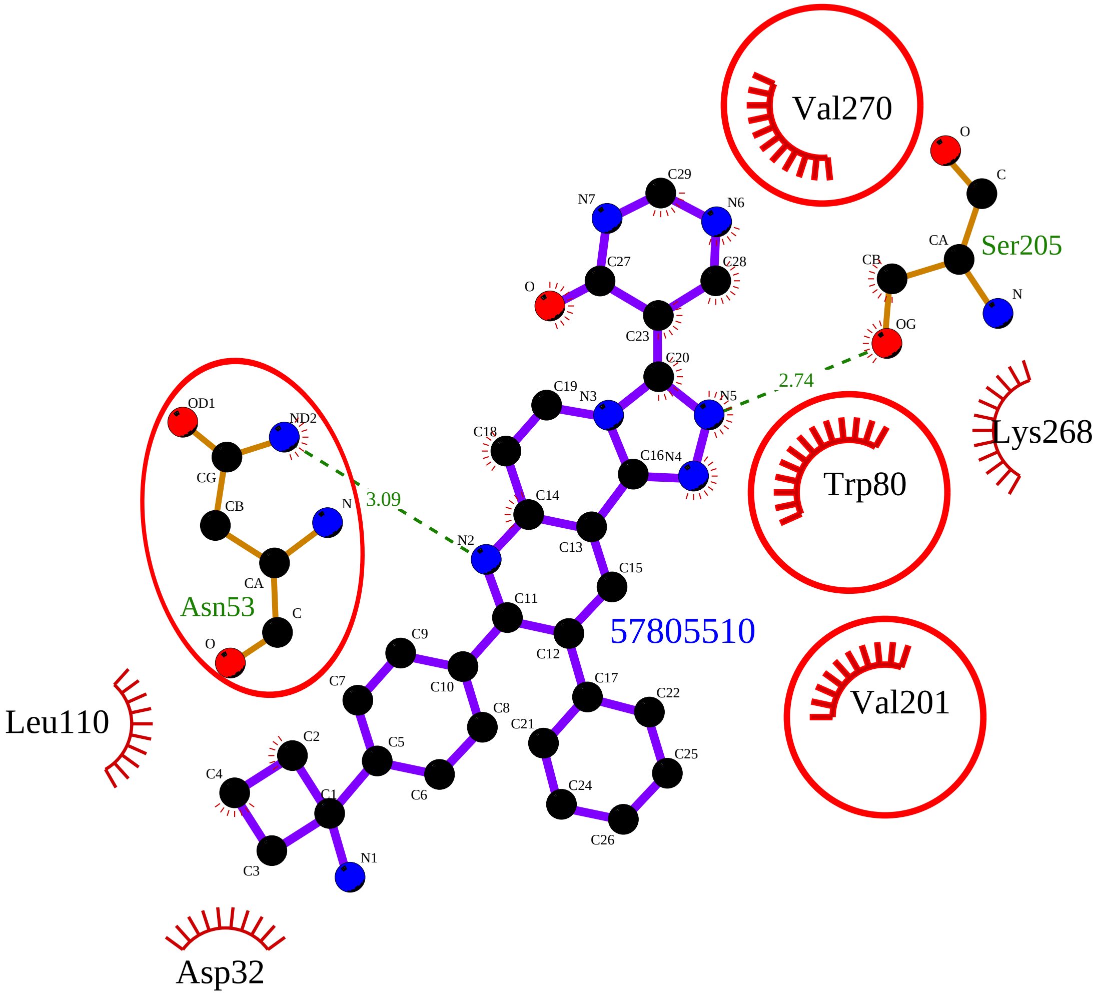
Val270
Ser205
2.74
Asn53
3.09
Lys268
Trp80
57805510
Val201
Leu110
Asp32

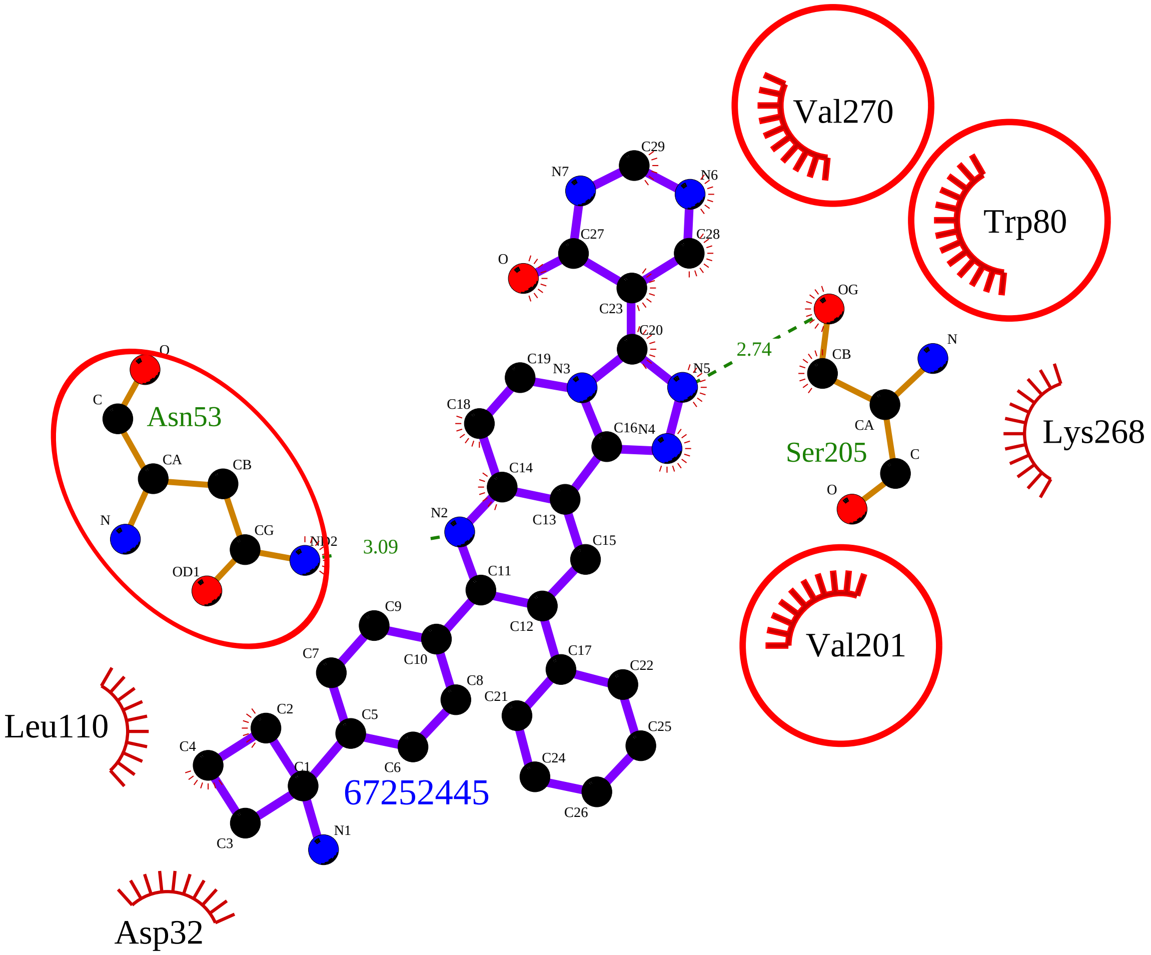
Val270
Trp80
Asn53
Ser205
2.74
Lys268
3.09
Val201
Leu110
67252445
Asp32

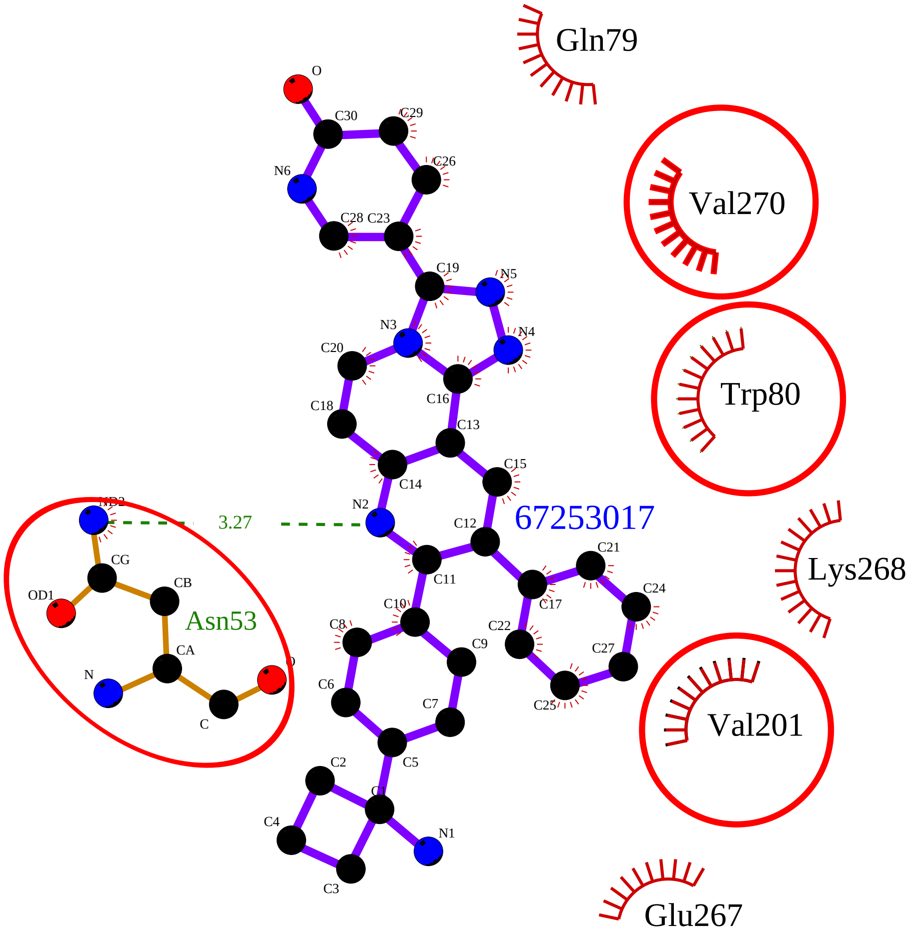

Gln79
Val270
Trp80
67253017
Lys268
Asn53
3.27
Val201
Glu267

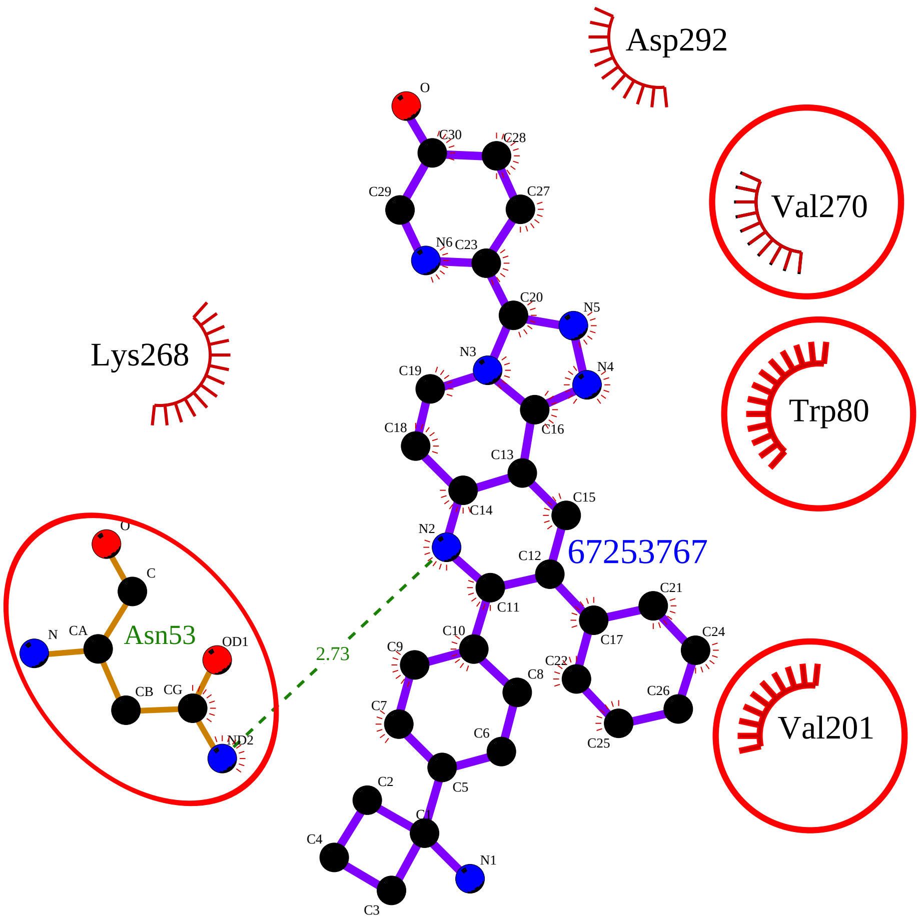
Asp292
Val270
Lys268
Trp80
67253767
Asn53
2.73
Val201
O
C30
C28
C29
C27
N6
C23
C20
N5
N3
N4
C19
C16
C18
C13
C15
C14
N2
C12
C11
C21
C24
C17
C10
C9
C8
C22
C26
C7
C6
C25
C5
C2
C1
C4
N1
C3
O
C
N
CA
OD1
CB
CG
ND2

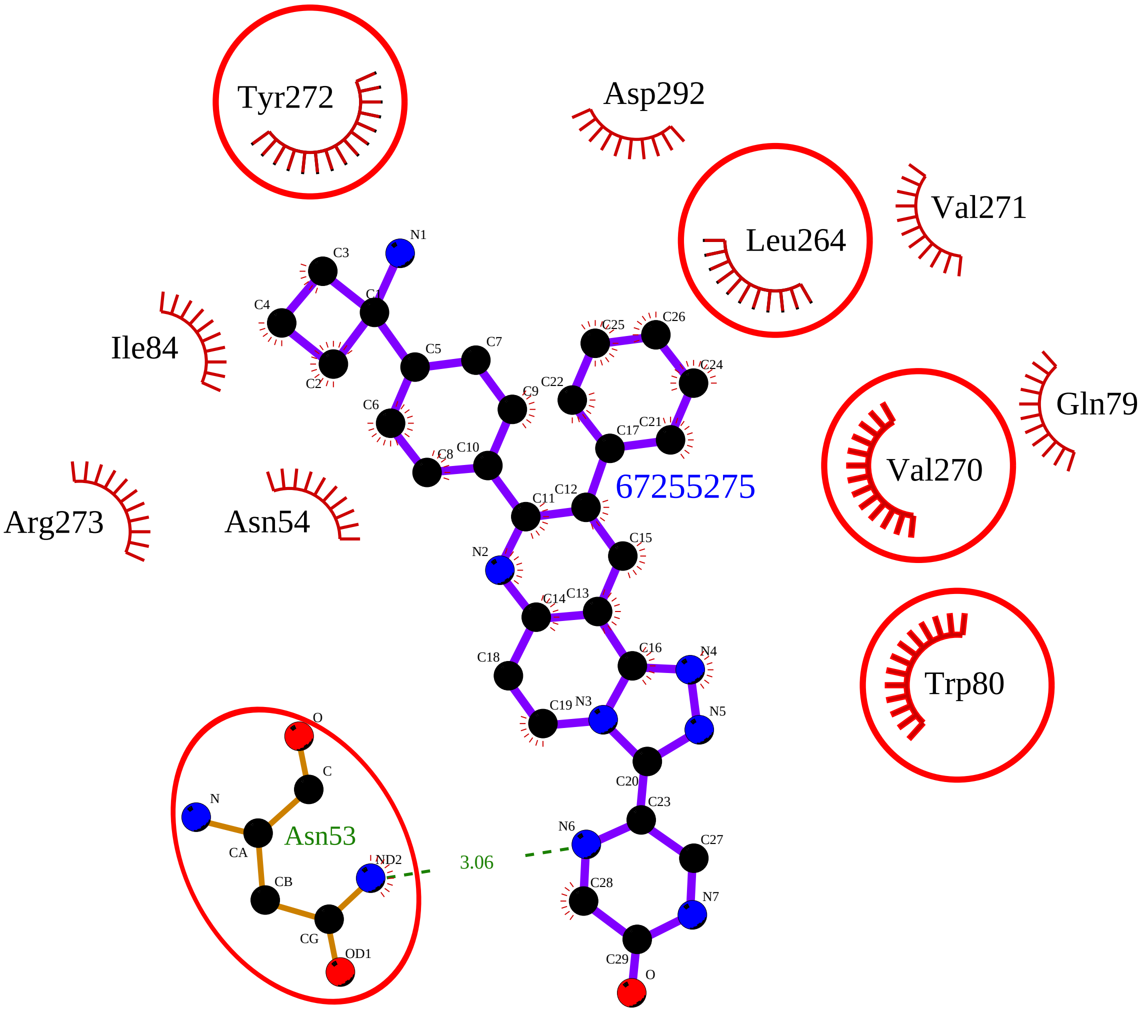

Tyr272
Asp292
Leu264
Val271
Ile84
Gln79
Val270
67255275
Arg273
Asn54
Trp80
Asn53
3.06
N1
C3
C4
C1
C2
C5
C7
C6
C8
C9
C10
C11
C12
C22
C25
C26
C24
C17
C21
N2
C15
C14
C13
C18
C16
N4
C19
N3
N5
C20
C23
N6
C27
C28
N7
C29
O
N
CA
C
CB
CG
ND2
OD1

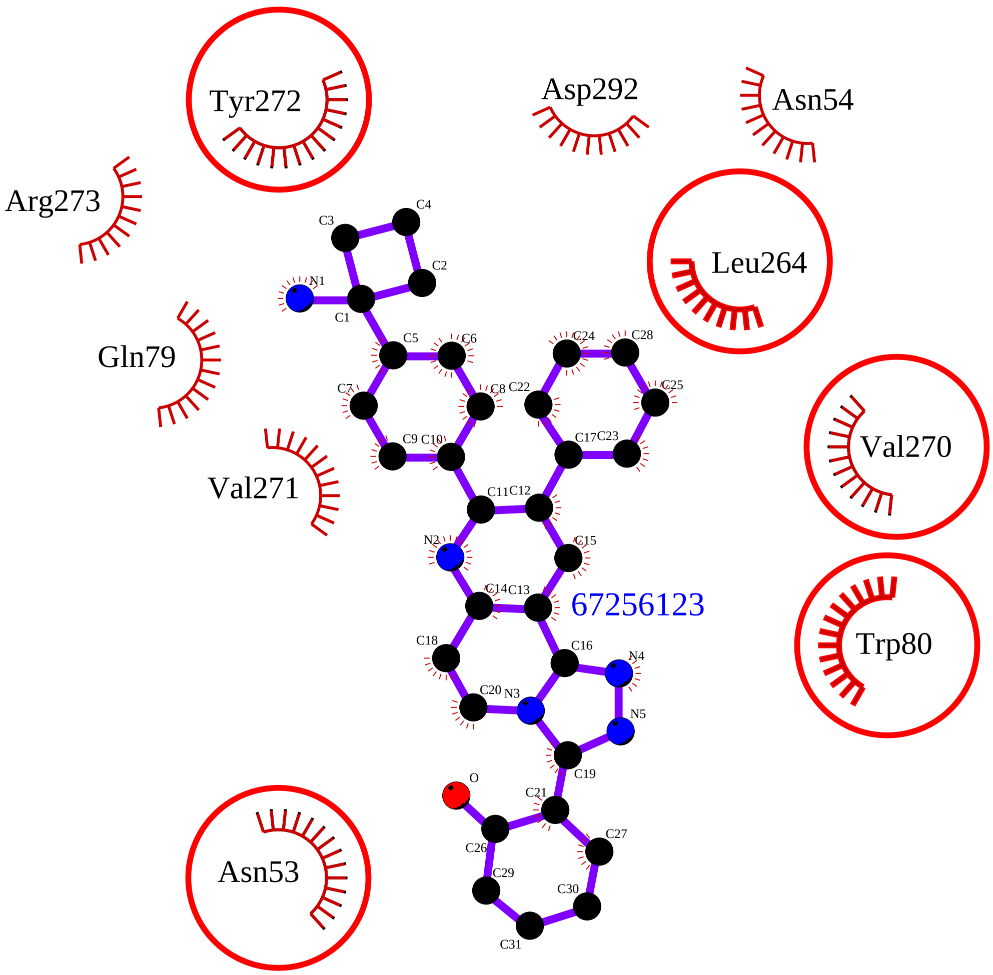

Tyr272
Asp292
Asn54
Arg273
Leu264
Gln79
Val270
Val271
Trp80
67256123
Asn53
N1
C1
C2
C3
C4
C5
C6
C7
C8
C9
C10
C11
C12
C13
C14
C15
C16
C17
C18
C19
C20
C21
C22
C23
C24
C25
C26
C27
C28
C29
C30
C31
N2
N3
N4
N5
O
