# Supplementary material for: Computational Insights into the Inhibitory Mechanism of Human AKT1 by an Orally Active Inhibitor, MK-2206
Source: PLoS One. 2014 Oct 17;9(10):e109705. doi: 10.1371/journal.pone.0109705 (PMC4201482; doi:10.1371/journal.pone.0109705)
Supplement: File S3 — R1R2-analogs of MK-2206. (PDF) [file pone.0109705.s003.pdf]

R_1R_2-analogs of MK-2206

Ligand-interaction plots of R_1R_2-analogs of MK-2206 are shown. The MK-2206 analogs' names are indicated by PubChem ID. The hydrogen bonds are shown as green-dashed lines with indicated bond length and the residues involved in hydrophobic interactions are shown as red arcs. The residues which are common to the first compound, the drug MK-2206 are encircled.

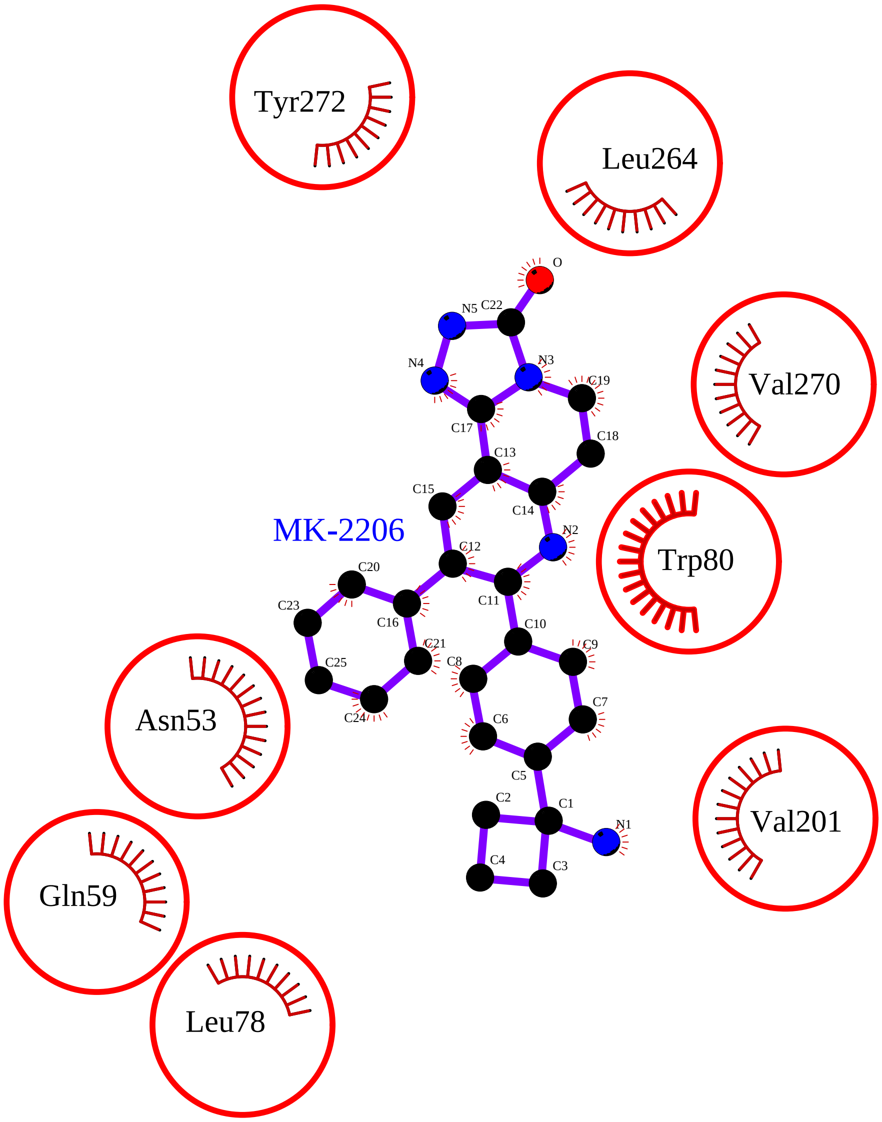
Tyr272
Leu264
O
N5
C22
N4
N3
C19
Val270
C17
C18
C13
C15
C14
Trp80
MK-2206
N2
C12
C20
C11
C23
C16
C10
C21
C9
C8
C25
Asn53
C7
C6
C24
C5
Val201
C2
C1
N1
C4
C3
Gln59
Leu78

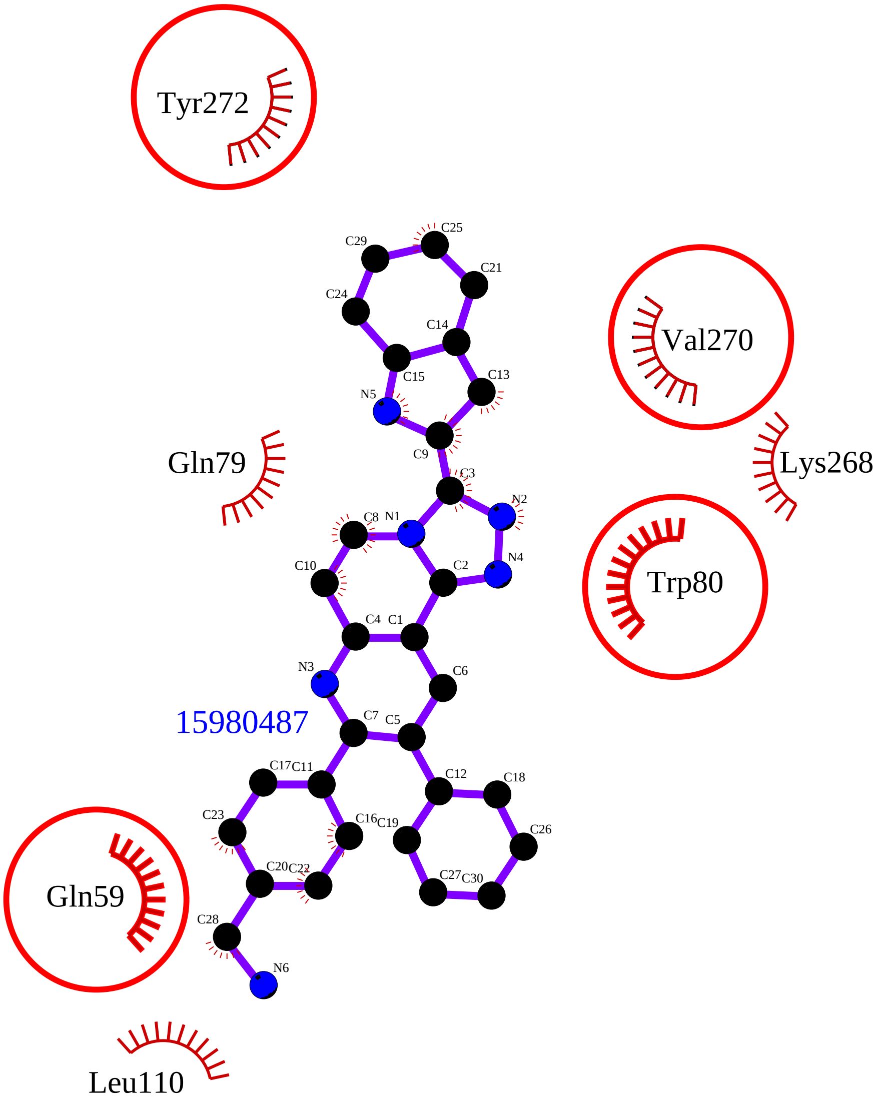
Tyr272
Val270
Lys268
Gln79
Trp80
Gln59
Leu110
15980487
C25
C29
C21
C24
C14
C15
C13
N5
C9
C3
N2
C8
N1
N4
C10
C2
C4
C1
N3
C6
C7
C5
C17
C11
C12
C18
C23
C16
C19
C26
C20
C22
C27
C30
C28
N6

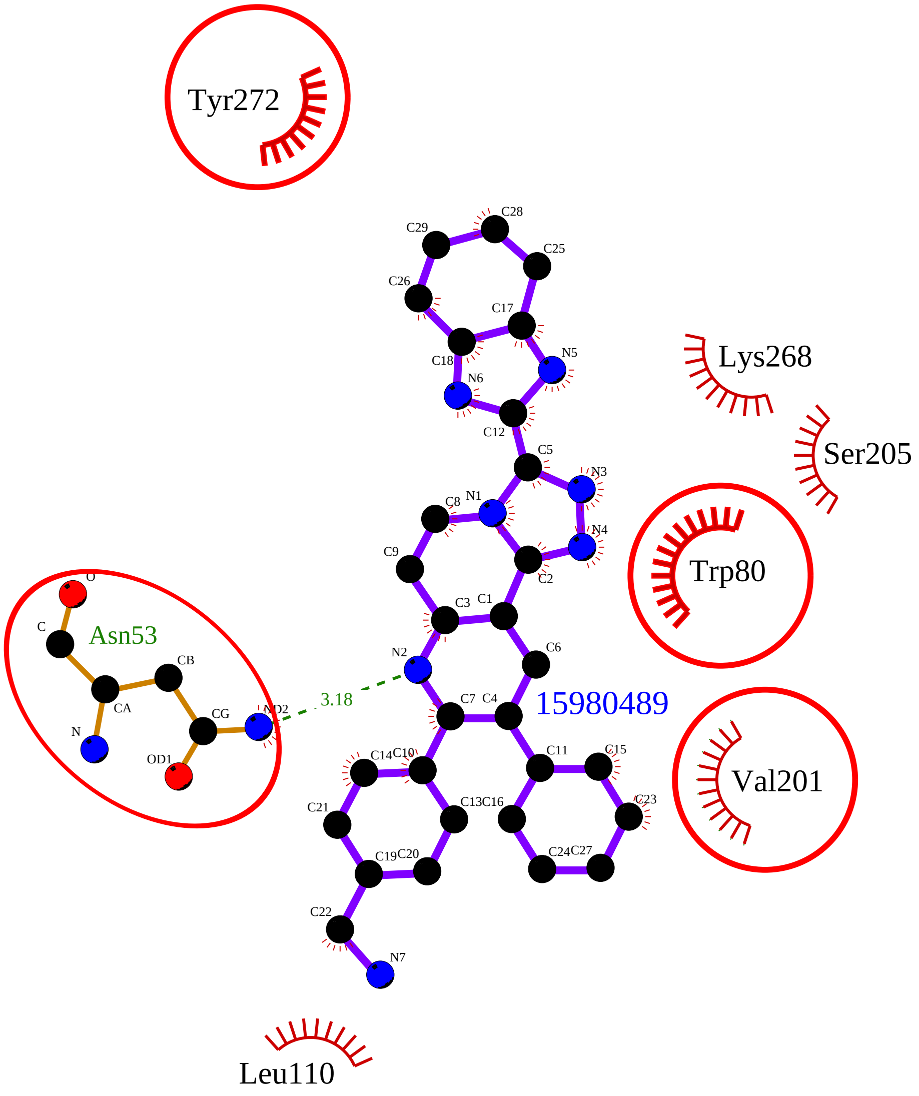
Tyr272
C28
C29
C25
C26
C17
N5
C18
N6
Lys268
C12
Ser205
C5
N3
C8
N1
N4
C9
C2
Trp80
O
C1
C3
C
Asn53
C6
CB
N2
3.18
C7
C4
15980489
CA
CG
ND2
N
C14
C10
C11
C15
OD1
Val201
C21
C13
C16
C23
C19
C20
C24
C27
C22
N7
Leu110

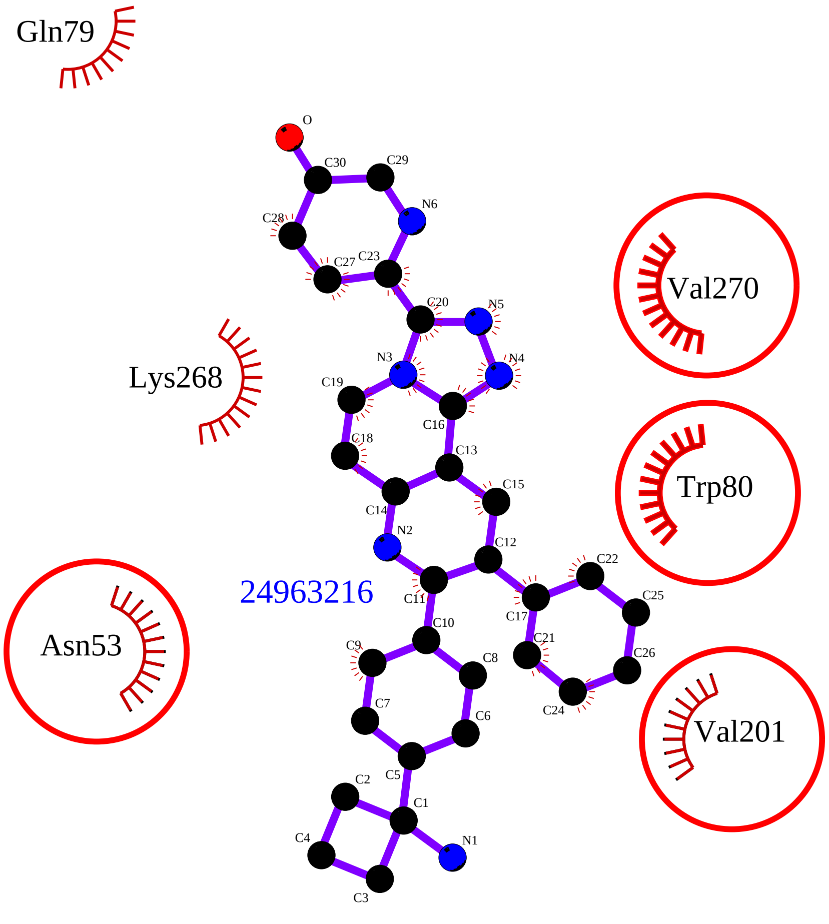

Gln79
O
C30
C29
N6
C28
C27
C23
C20
N5
Val270
Lys268
N3
N4
C19
C16
C18
C13
Trp80
C15
C14
N2
C12
C22
24963216
C11
C17
C25
Asn53
C10
C21
C9
C8
C26
C24
C7
C6
Val201
C5
C2
C1
C4
N1
C3

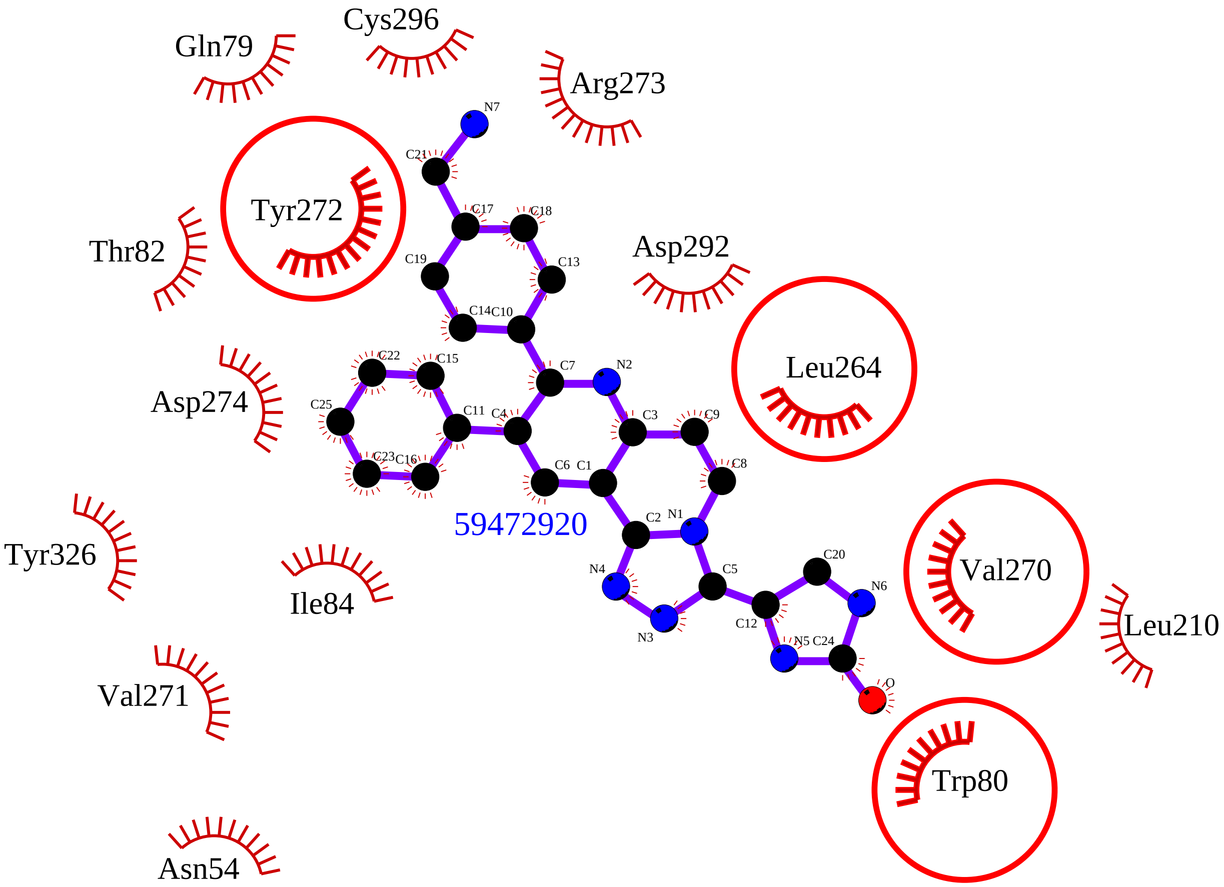

Gln79
Cys296
Arg273
N7
C21
Tyr272
C17
C18
Thr82
C19
C13
Asp292
C14C10
Leu264
C22
C15
C7
N2
Asp274
C25
C11
C4
C3
C9
C23C16
C6
C1
C8
59472920
C2
N1
Tyr326
Ile84
N4
C5
C20
Val270
N6
N3
C12
N5 C24
Leu210
Val271
O
Trp80
Asn54

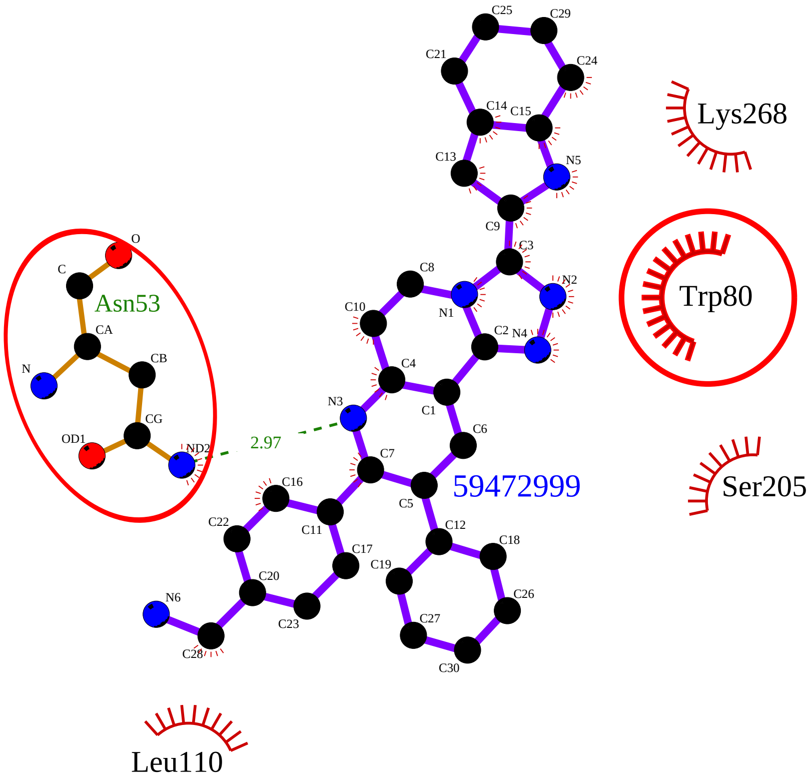
C25
C29
C21
C24
Lys268
C14
C15
C13
N5
C9
C3
O
C
Asn53
C8
N2
Trp80
C10
N1
C2
N4
CA
CB
C4
N
N3
C1
CG
C6
OD1
ND2
2.97
C7
C16
C5
59472999
Ser205
C22
C11
C12
C18
C17
C19
C20
C26
N6
C23
C27
C28
C30
Leu110

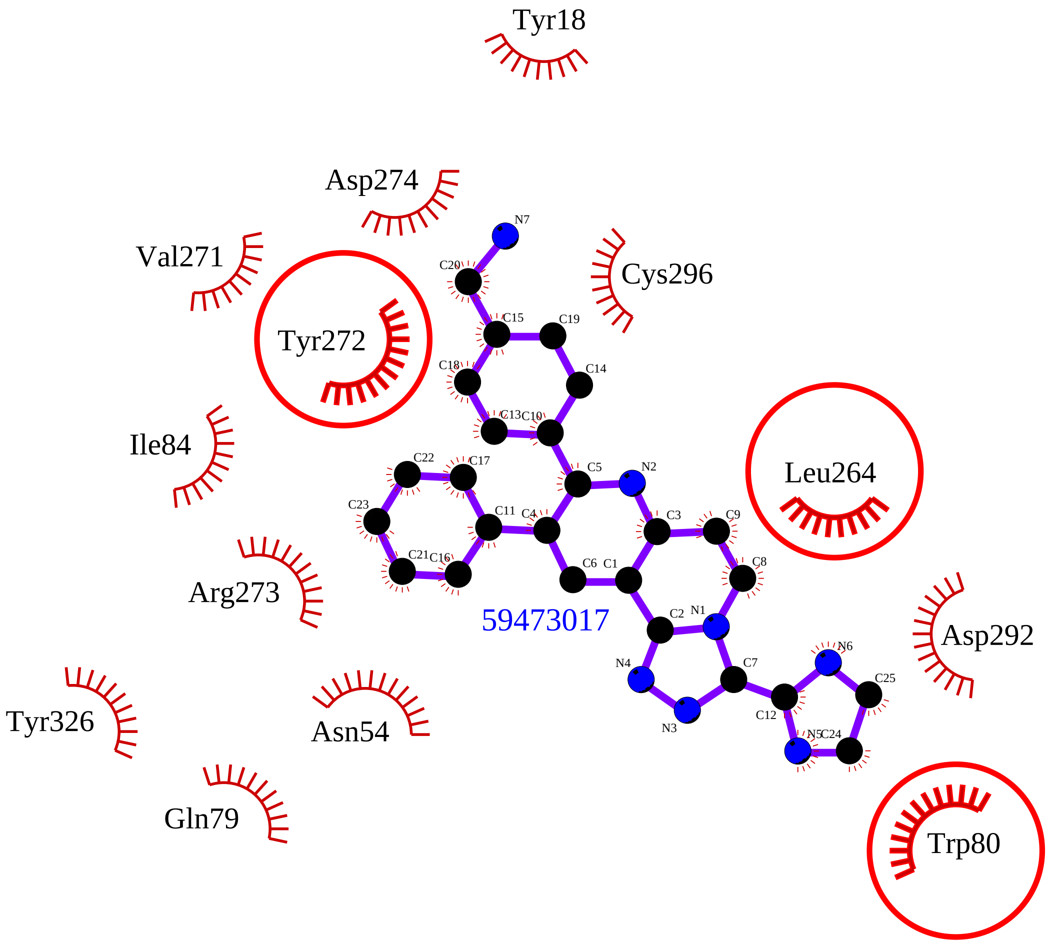

Tyr18
Asp274
Val271
Cys296
Tyr272
Ile84
Leu264
Arg273
59473017
Asp292
Tyr326
Asn54
Gln79
Trp80
N7
C20
C15
C19
C18
C14
C13
C10
C22
C17
C5
N2
C23
C11
C4
C3
C9
C21
C16
C6
C1
C8
C2
N1
N4
C7
N6
C25
N3
C12
N5
C24

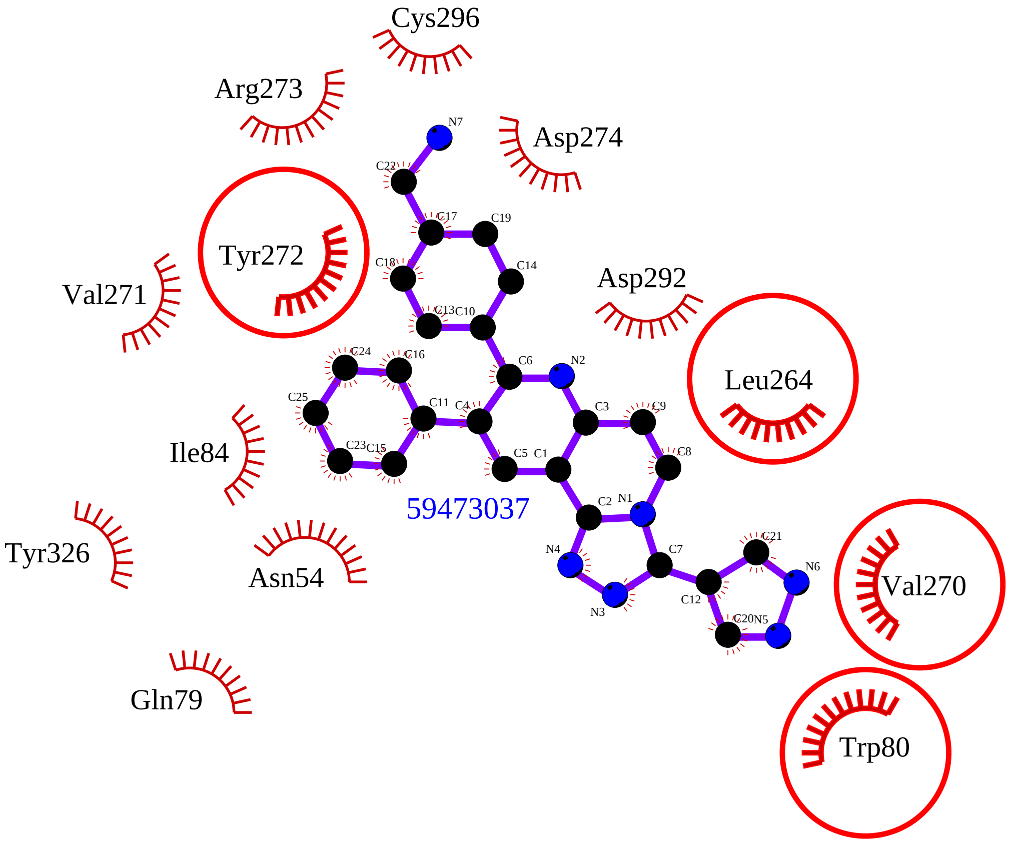
Cys296
Arg273
Asp274
Tyr272
Val271
Asp292
Leu264
Ile84
Tyr326
Asn54
Gln79
Val270
Trp80
59473037
N7
C22
C17
C19
C18
C14
C13
C10
C24
C16
C6
N2
C25
C11
C4
C3
C9
C23
C15
C5
C1
C8
C2
N1
N4
C7
C21
N6
N3
C12
C20
N5

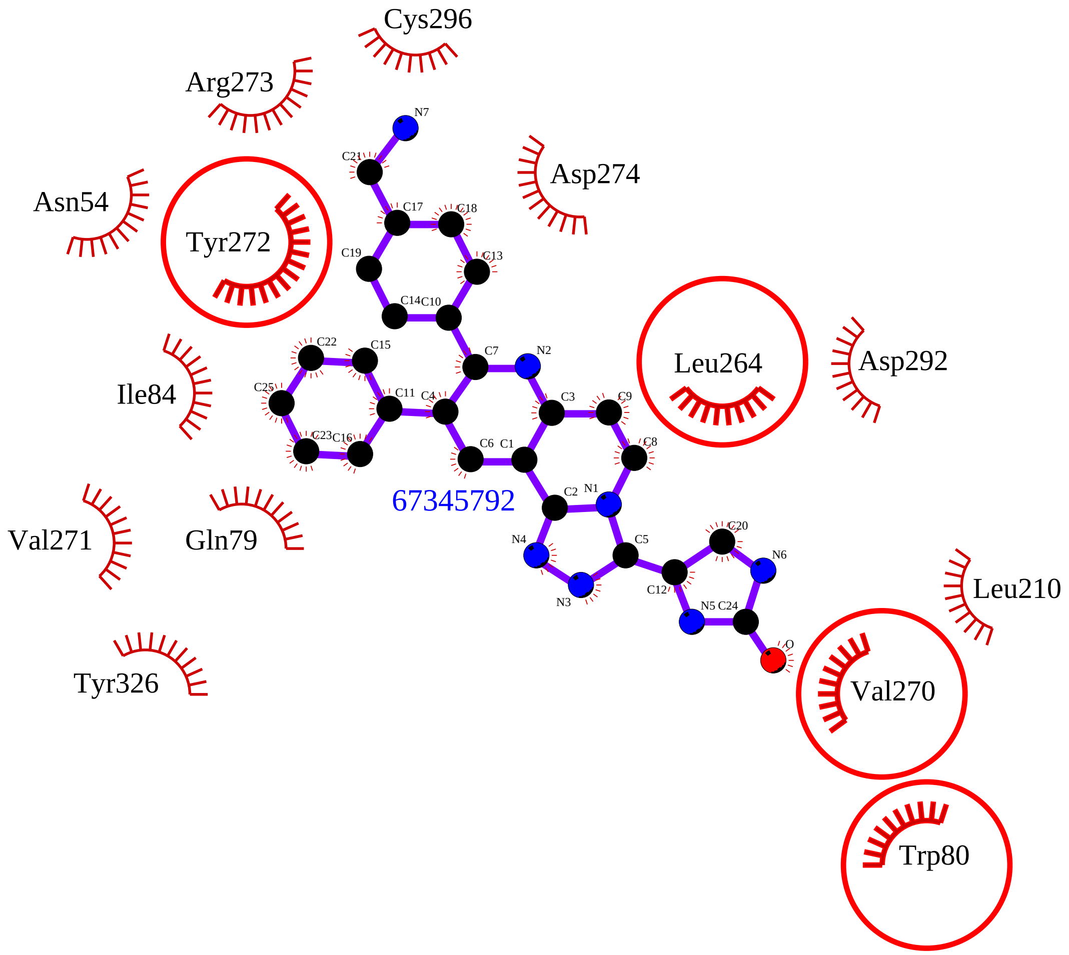

Cys296
Arg273
Asn54
Tyr272
Asp274
Leu264
Asp292
Ile84
Val271
Gln79
Tyr326
67345792
Leu210
Val270
Trp80

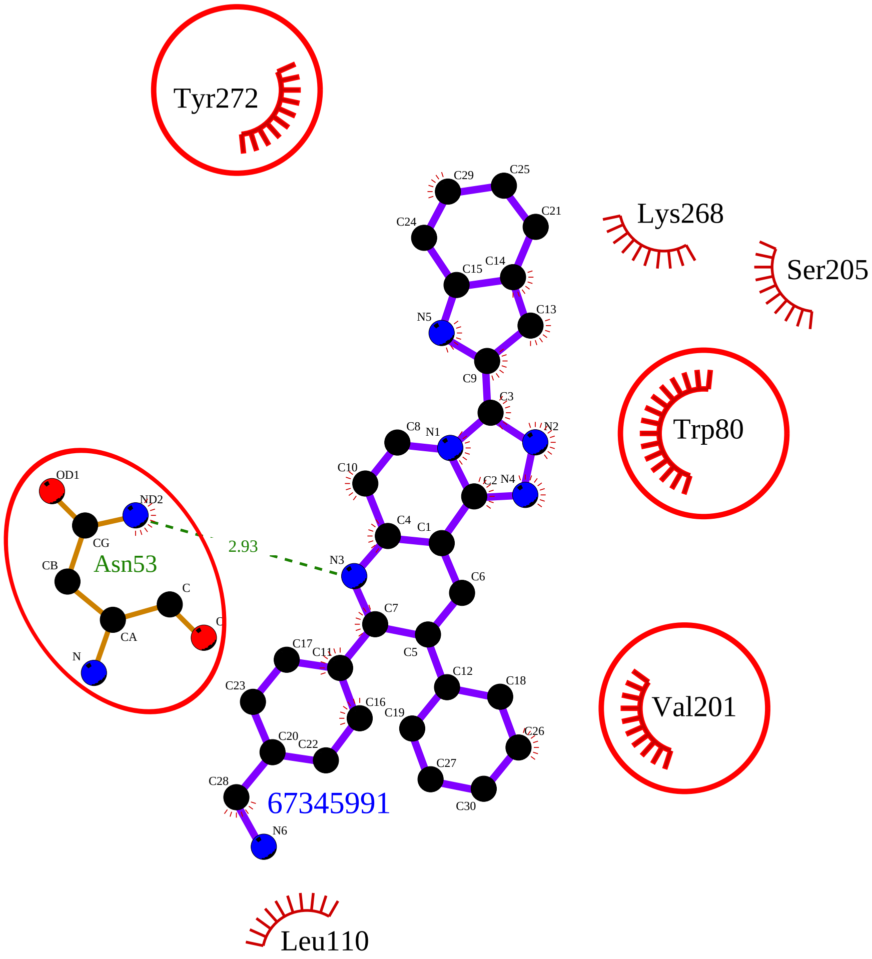
Tyr272
Lys268
Ser205
Trp80
Val201
Leu110
Asn53
2.93
67345991
OD1
ND2
CG
CB
CA
C
N
C29
C25
C24
C21
C15
C14
N5
C13
C9
C3
N2
C8
N1
C10
C2
N4
C4
C1
N3
C6
C7
C5
C17
C11
C23
C16
C12
C18
C19
C26
C20
C22
C27
C30
C28
N6

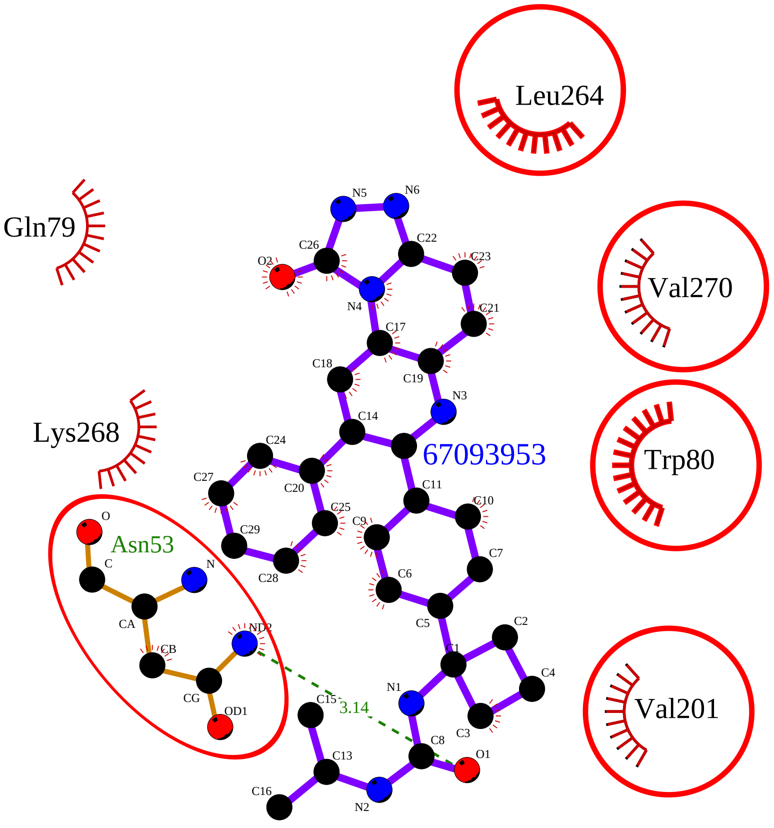

Leu264
Gln79
Val270
Lys268
Trp80
67093953
Asn53
3.14
Val201

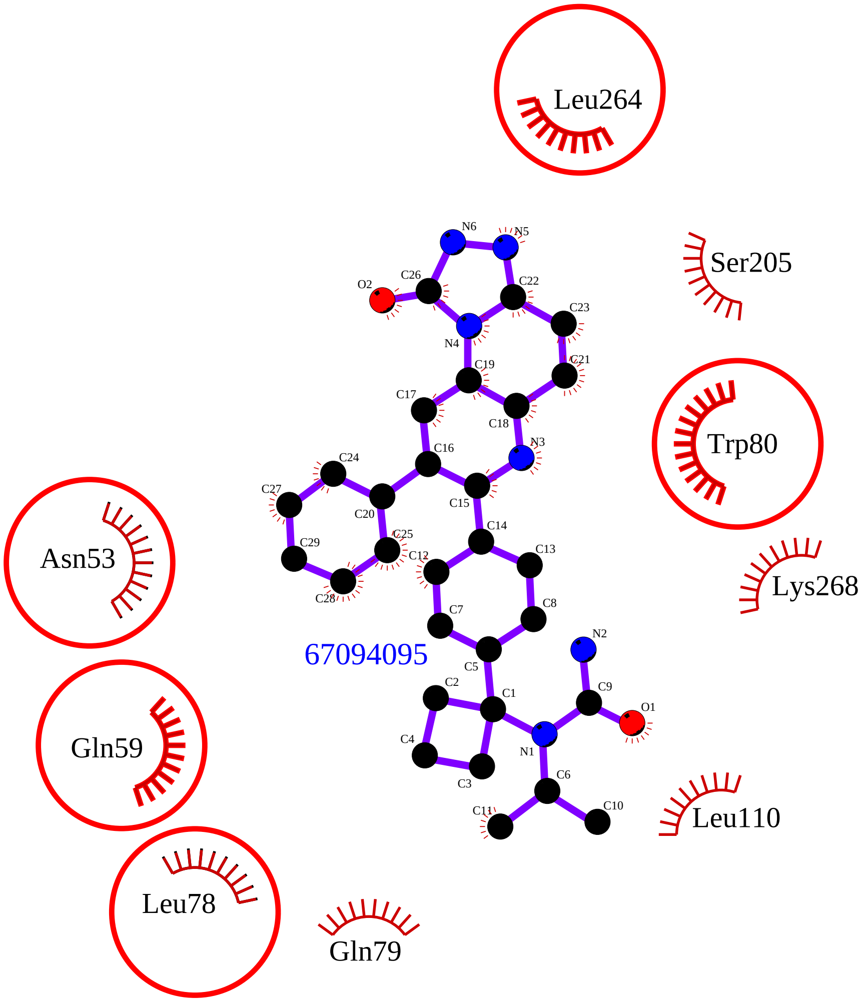

Leu264
Ser205
Trp80
Lys268
Asn53
Gln59
Leu78
Gln79
Leu110
67094095
N6
N5
O2
C26
C22
C23
N4
C19
C21
C17
C18
N3
C16
C24
C27
C20
C15
C14
C25
C29
C12
C13
C28
C8
C7
N2
C5
C9
C2
C1
O1
C4
N1
C3
C6
C11
C10

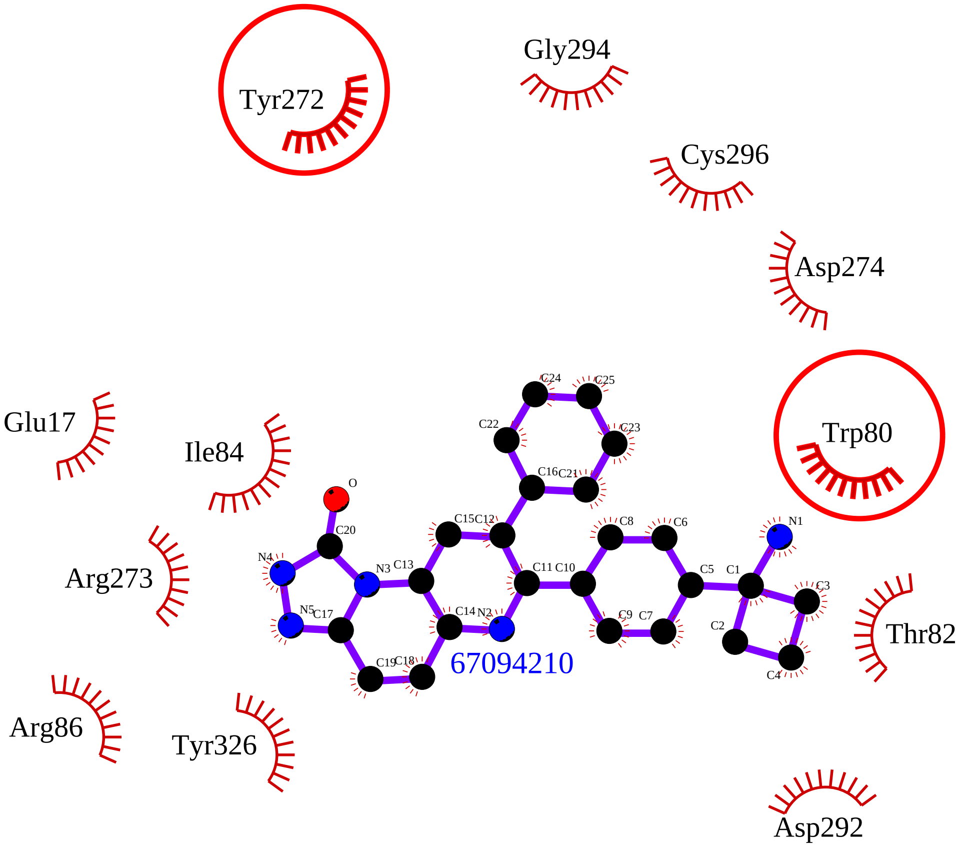
Tyr272
Gly294
Cys296
Asp274
Glu17
Ile84
Trp80
Arg273
Thr82
Arg86
Tyr326
Asp292
67094210
O
C20
N4
N3
C13
N5
C17
C15
C12
C16
C21
C22
C24
C25
C23
C11
C10
C8
C6
C5
C1
N1
C3
C2
C4
C14
N2
C19
C18
C9
C7
