# Supplementary material for: Computational Insights into the Inhibitory Mechanism of Human AKT1 by an Orally Active Inhibitor, MK-2206
Source: PLoS One. 2014 Oct 17;9(10):e109705. doi: 10.1371/journal.pone.0109705 (PMC4201482; doi:10.1371/journal.pone.0109705)
Supplement: Table S1 — The binding strength of structural analogs of MK-2206 to human AKT1 given by various scores are listed in the table. These are classified as R1-, R2- and R1R2- structural analogs of MK-2206 as described in Materials and Method section. (DOC) [file pone.0109705.s004.doc]

Table S1. The binding strength of structural analogs of MK-2206 to human AKT1 given by various scores are listed in the table. These are classified as R_1_-, R_2_- and R_1_R_2_- structural analogs of MK-2206 as described in Materials and Method section.

| **CID** | **Binding energy** | **pK_d_** | **Dock (Grid) score** |
| --- | --- | --- | --- |
| **R_1_ Structural analogs (6)** | | | |
| MK-2206 | -8.83 | 6.47 | -26.55 |
| 15979152 | -8.24 | 6.04 | -30.16 |
| 44556180 | -8.78 | 6.44 | -32.77 |
| 44556292 | -8.97 | 6.57 | -30.21 |
| 67979193 | -9.95 | 7.29 | -39.23 |
| 68299597 | -8.80 | 6.45 | -30.25 |
| 70864634 | -8.88 | 6.51 | -32.00 |
| **R_2_ Structural analogs (12)** | | | |
| 24965693 | -9.80 | 7.20 | -19.00 |
| 24965696 | -9.00 | 6.60 | -31.00 |
| 24966396 | -9.90 | 7.30 | -31.00 |
| 24966749 | -8.60 | 6.30 | -30.00 |
| 24966751 | -9.90 | 7.20 | -31.00 |
| 24966752 | -8.60 | 6.30 | -34.00 |
| 57805510 | -8.30 | 6.10 | -23.00 |
| 67252445 | -8.30 | 6.10 | -23.00 |
| 67253017 | -8.90 | 6.50 | -34.00 |
| 67253767 | -8.70 | 6.40 | -20.00 |
| 67255275 | -9.90 | 7.20 | -28.00 |
| 67256123 | -10.00 | 7.40 | -23.00 |
| **R_1_R_2_ Structural analogs (12)** | | | |
| 15980487 | -9.29 | 6.81 | -37.93 |
| 15980489 | -9.02 | 6.61 | -39.94 |
| 24963216 | -8.78 | 6.44 | -28.40 |
| 59472920 | -9.42 | 6.91 | -41.92 |
| 59472999 | -9.06 | 6.64 | -35.94 |
| 59473017 | -9.36 | 6.86 | -35.56 |
| 59473037 | -9.34 | 6.85 | -39.79 |
| 67345792 | -9.43 | 6.91 | -40.22 |
| 67345991 | -9.27 | 6.79 | -33.87 |
| 67093953 | -9.57 | 7.02 | -31.53 |
| 67094095 | -9.08 | 6.66 | -34.57 |
| 67094210 | -9.59 | 7.03 | -35.53 |
